# Nonessential tRNA and rRNA modifications impact the bacterial response to sub-MIC antibiotic stress

**DOI:** 10.1093/femsml/uqac019

**Published:** 2022-09-14

**Authors:** Anamaria Babosan, Louna Fruchard, Evelyne Krin, André Carvalho, Didier Mazel, Zeynep Baharoglu

**Affiliations:** Département Génomes et Génétique, Institut Pasteur, UMR3525, CNRS, Unité Plasticité du Génome Bactérien, 25 rue du Dr Roux 75015 Paris, France; Département Génomes et Génétique, Institut Pasteur, UMR3525, CNRS, Unité Plasticité du Génome Bactérien, 25 rue du Dr Roux 75015 Paris, France; Sorbonne Université, Collège Doctoral, F-75005 Paris, France; Département Génomes et Génétique, Institut Pasteur, UMR3525, CNRS, Unité Plasticité du Génome Bactérien, 25 rue du Dr Roux 75015 Paris, France; Département Génomes et Génétique, Institut Pasteur, UMR3525, CNRS, Unité Plasticité du Génome Bactérien, 25 rue du Dr Roux 75015 Paris, France; Sorbonne Université, Collège Doctoral, F-75005 Paris, France; Département Génomes et Génétique, Institut Pasteur, UMR3525, CNRS, Unité Plasticité du Génome Bactérien, 25 rue du Dr Roux 75015 Paris, France; Département Génomes et Génétique, Institut Pasteur, UMR3525, CNRS, Unité Plasticité du Génome Bactérien, 25 rue du Dr Roux 75015 Paris, France

**Keywords:** RNA modifications, *Vibrio cholerae*, sub-MIC antibiotics, antibiotic resistance, bacterial stress responses, differential translation

## Abstract

Antimicrobial resistance develops as a major problem in infectious diseases treatment. While antibiotic resistance mechanisms are usually studied using lethal antibiotic doses, lower doses allowing bacterial growth are now considered as factors influencing the development and selection of resistance. Starting with a high-density Tn insertion library in *Vibrio cholerae* and following its evolution by TN-seq in the presence of subinhibitory concentrations of antibiotics, we discovered that RNA modification genes can have opposite fates, being selected or counter-selected. We, thus have undertaken the phenotypic characterization of 23 transfer RNA (tRNA) and ribosomal RNA (rRNA) modifications deletion mutants, for which growth is globally not affected in the absence of stress. We uncover a specific involvement of different RNA modification genes in the response to aminoglycosides (tobramycin and gentamicin), fluoroquinolones (ciprofloxacin), β-lactams (carbenicillin), chloramphenicol, and trimethoprim. Our results identify t/rRNA modification genes, not previously associated to any antibiotic resistance phenotype, as important factors affecting the bacterial response to low doses of antibiotics from different families. This suggests differential translation and codon decoding as critical factors involved in the bacterial response to stress.

## Introduction

Antibiotic overuse and misuse contribute to antimicrobial resistance (AMR), via selective pressure exerted by treatment during infection, but also in the environment where gradients of antibiotics are found in soil and water, the natural reservoir of many bacteria among which *Vibrio*. AMR is increasingly associated with life in the aquatic environment, particularly in aquaculture farms, where several bacterial species coexist. A World Health Organization report on AMR in enteric pathogens states that “consideration must be given to the relationship of *Vibrio* with the environment” to understand AMR development (Sack et al. 2001). Most studies address the bacterial response to lethal antibiotic concentrations and the effect of gene mutations on antibiotic resistance. Meanwhile, in their environments, bacteria encounter sub-minimal inhibitory concentrations (sub-MICs) of antibiotics (Chow et al. 2021), which are stressors, and can lead to transient phenotypic tolerance to high doses of antibiotics (Andersson and Hughes 2014). Thus, characterization of the bacterial responses to such stress and its impact on resistance/tolerance, need to be comprehensively clarified.

We have previously demonstrated that several pathways identified for the response to antibiotic stress in *Vibrio cholerae* are paradigmatic for other bacterial pathogens (Baharoglu and Mazel 2011, Baharoglu et al. 2013). Using sub-MIC antibiotics, we aimed at characterizing, which bacterial responses were triggered and allowed the cells to grow and survive, and we asked whether the identified processes also impact bacterial phenotypes at lethal concentrations of the same antibiotics. Our results point to a central role of transfer RNA (tRNA) and ribosomal RNA (rRNA) modifications in the response to sub-MIC antibiotic stress, suggesting that RNA modification profiles and translation may be modified in bacteria by stress.

Evolution of resistance requires genetic diversity in populations, yet nongenetic phenotypic diversity can also contribute. One process generating phenotypic diversity is translation, with an average error rate of 10^−4^ and up to 10^−3^ substitutions per position under stress conditions (Kramer and Farabaugh 2007). Translation errors cause protein misfolding (Nedialkova and Leidel 2015, Liu 2020), aggregation and proteotoxic stress (Drummond and Wilke 2009). Translation errors can also provide transient increase in fitness (Samhita et al. 2020), offering cells the necessary time to acquire beneficial genetic mutations (Whitehead et al. 2008) and to eliminate deleterious ones (Bratulic et al. 2017), as it was observed upon oxidative stress (Netzer et al. 2009) and proteotoxic stress (Evans et al. 2019).

Codon decoding efficiency can impact translation speed or translation accuracy at specific mRNAs/codons, and proteome diversity (Kramer and Farabaugh 2007). Differences in decoding and reading frame maintenance have already been linked with the presence or absence of certain RNA modifications (Baudin-Baillieu and Namy 2021, Valadon and Namy 2021). In particular, methylation at specific positions in rRNA stabilizes the binding of initiator tRNA to the ribosome at the start codon (Burakovsky et al. 2012), and several rRNA methylation factors have been linked to aminoglycoside (AG) resistance (Doi and Arakawa 2007, Dunkle et al. 2014). Regarding tRNA modifications, more than 80 have been described in bacteria (de Crecy-Lagard and Jaroch 2021). They can be involved in tRNA stability (Motorin and Helm 2010), abundance (Kramer and Farabaugh 2007), decay (Kimura and Waldor 2019, Hughes et al. 2020), and affinity for the ribosome (Noguchi et al. 1982). While some tRNA modification genes are essential, (e.g. *trmD* and *tadA*), in many cases their deletion does not confer any visible phenotype to the unstressed cells [de Crecy-Lagard and Jaroch 2021; Table S1 (Supporting Information) and references therein). Few studies address the exact physiological roles of nonessential rRNA (Zou et al. 2018, Georgeson and Schwartz 2021) and tRNA modifications in bacterial stress response phenotypes (Vecerek et al. 2007, Toh and Mankin 2008, Aubee et al. 2016, Chionh et al. 2016, Hou et al. 2017, Thongdee et al. 2019), reviewed in de Crecy-Lagard and Jaroch (2021).

In the present study, we reveal the involvement of various RNA modification genes, in the response to antibiotics. The RNA modification genes identified here are different from previously described modifications conferring antibiotic resistance. We show that their inactivation confers, not resistance, but increased or decreased fitness in presence of antibiotic stress.

## Results

### TN-seq identifies rRNA and tRNA modification genes involved in the response to sub-MIC TOB and CIP in *V. cholerae*

**Figure 1:**
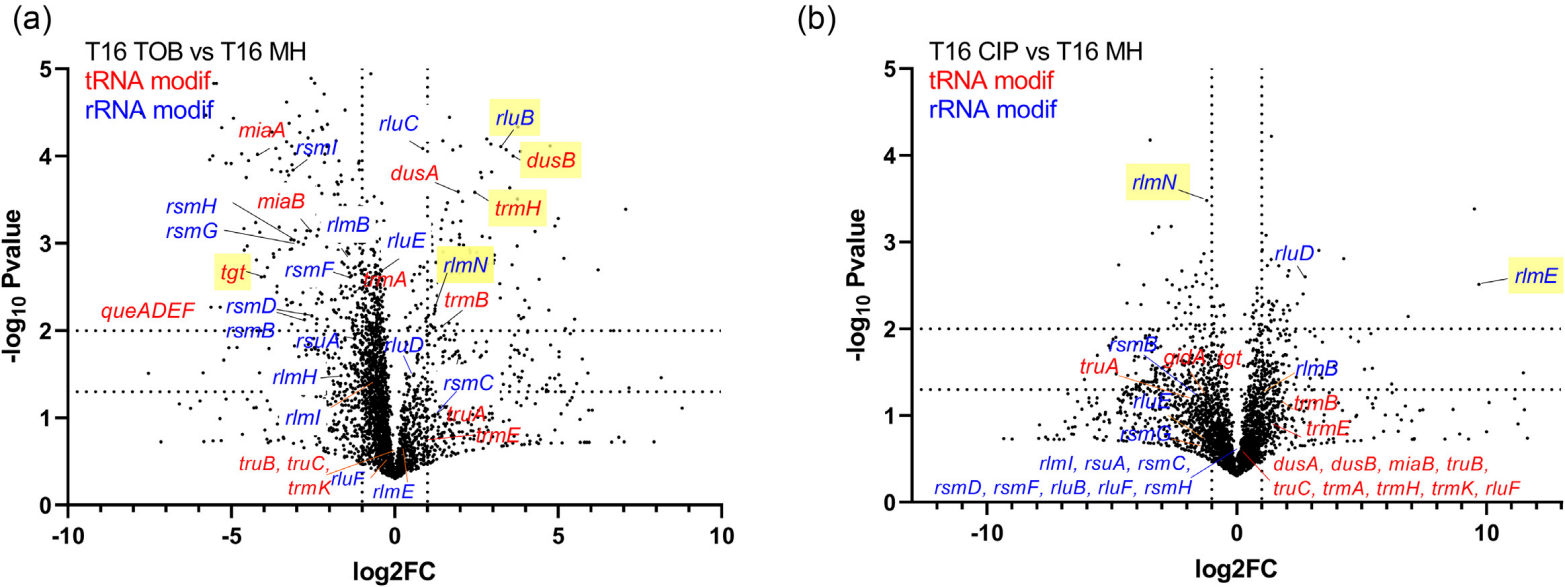
TN-seq identifies rRNA and tRNA modification genes affecting fitness of *V. cholerae* in the presence of sub-MIC TOB and CIP. tRNA modification genes are indicated in red. rRNA modification genes are indicated in blue. *rlmN* modifies both tRNAs and rRNA. Volcano plots show genes for which the number of transposon inactivation is increased (beneficial) or decreased (detrimental) after 16 generations of growth, compared to growth without antibiotics. (A) TOB 50% of the MIC, (B) CIP 50% of the MIC. *x*-axis represents log2 fold change of the number of transposon reads associated with gene inactivations, detected after 16 generations in the indicated antibiotic versus nontreated condition. The *y*-axis represents the negative log_10_*P-value*. Gene inactivations, which show the strongest antibiotic specific effects are highlighted in yellow. Dotted lines in the *y*-axis indicate *P*-values of .05 (lower line) and .01 (upper line). Dotted lines in the *x*-axis represent 2-fold decrease (left) and 2-fold increase (right).

**Figure 2:**
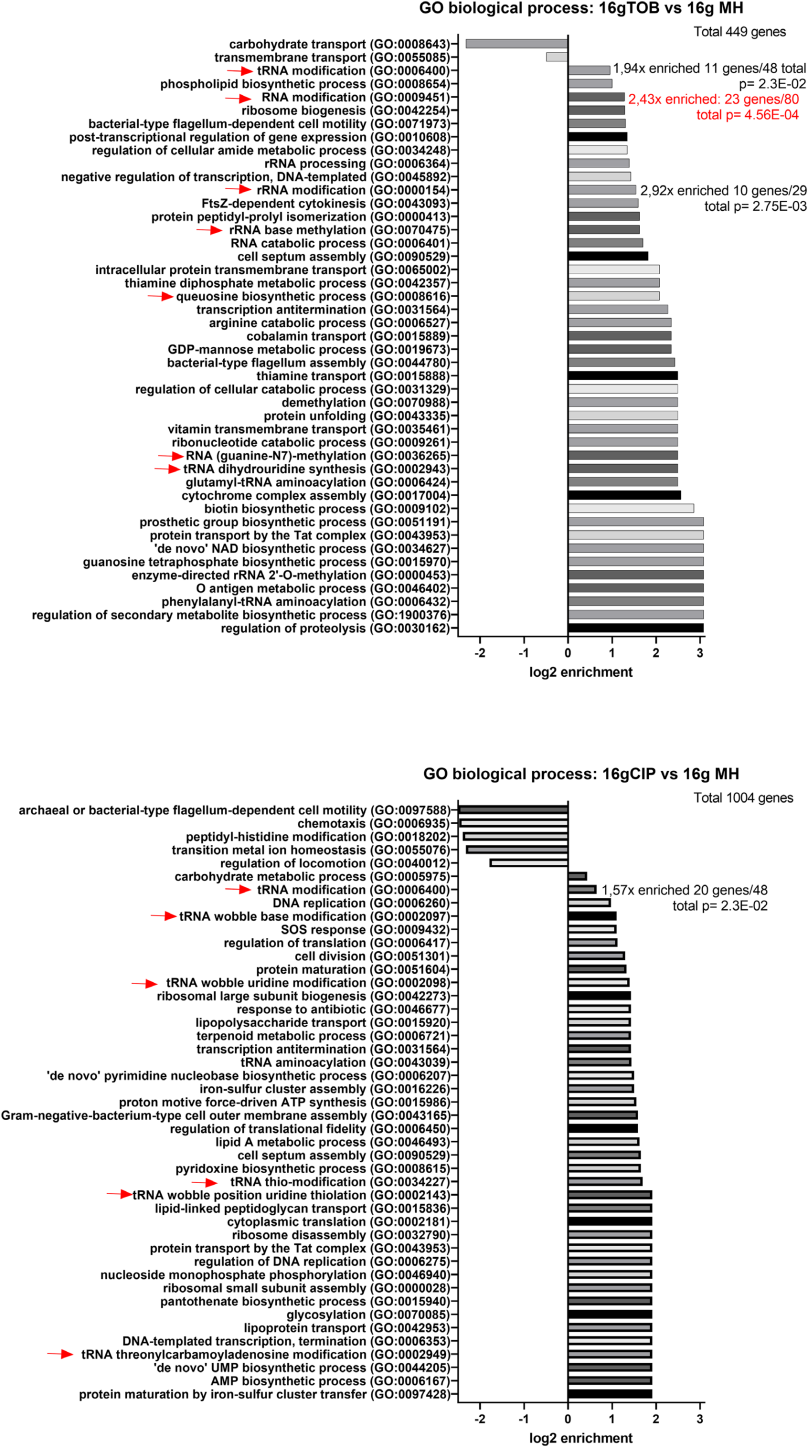
GO analysis for TN-seq data. The analyzed lists were for each antibiotic (TOB/CIP), genes (plotted on Fig. 1) with at least 2-fold change in TN-seq data at 16 generations compared to nontreated condition, and with an adjusted (Bonferroni correction) *P*-value < .05. Red arrows indicate RNA modification processes. The total number of uploaded genes list to be analyzed were 449 genes for TOB and 1004 genes for CIP. The reference gene list was *V. cholerae* (all genes in database), 3782 genes. Annotation version: PANTHER Overrepresentation Test (Released 20220712). GO Ontology database DOI: 10.5281/zenodo.6399963 Released 2022-03-22. Only the results with the Fold Enrichment of the most specific categories are shown, the detailed results are shown in Table S2 (Supporting Information).

Using TN-seq in *V. cholerae*, we searched for genes that are important for growth in the presence of sub-MICs of antibiotics targeting the ribosome [TOB: tobramycin belonging to AGs), or DNA (CIP: ciprofloxacin belonging to fluoroquinolones (FQs)]. We constructed large transposon inactivation libraries in *V. cholerae* as previously performed (Negro et al. 2019), and we subjected them to growth without or with antibiotics at 50% of the minimal inhibitory concentration (MIC), during 16 generations. After sequencing and bioinformatics analysis of the regions flanking the transposon, we identified genes where reads associated to detected transposon insertions increase or decrease. Loss of detected insertions in a specific gene generally means that the inactivation of this gene is detrimental in the tested condition, while enrichment means that the inactivation is beneficial. In some cases, transposon insertion in one gene may also lead to differential expression of downstream genes. In this study, we searched for genes that are important only during sub-MIC treatment. We, thus compared insertion counts after 16 generations in sub-MIC antibiotics (TOB or CIP) to those after 16 generations without antibiotics (Fig. 1 and Table 1; Table S1, Supporting Information). Genes having a significant impact on fitness (insertions enriched or lost) in the nontreated condition are thus not included in our analysis. Gene ontology (GO) analysis was performed for genes with more than 2-fold changes in the number of insertions and with a significant *P*-value (Fig. 2; Table S2, Supporting Information). For both antibiotics, we found common and antibiotic specific RNA modification genes whose number of reads was impacted, suggesting that their inactivation was either beneficial or detrimental for growth in the presence of the sub-MIC antibiotic. Results show a 2.43x enrichment (*P* = 4.56×10^−4^) of RNA modification genes (GO: 0009451) with 23 genes identified out of a total of 80 for TOB and 1.57x enrichment (*P* = 2.3×10^−2^) for tRNA modification genes (GO: 0006400) with 20 identified out of a total of 48 for CIP. As expected, other categories identified include expected categories such as proteolysis for TOB or DNA replication/SOS response for CIP (Fig. 2).

**Table 1.** Phenotypes associated to RNA modification genes identified by TN-seq. “N/A” means: not selected for further study. NS: nonsignificant *P-*value.

*Gene*	Function	Known physiological phenotypes in literature	Known effects on translation in literature	TN-seq fold change (T16 antibiotic/T16MH)—this study
**tRNA modification**				
*tgt*	tRNA-guanine transglycosylase GUN anticodon tRNAs	No significant biological difference but defect in stationary phase viability (Noguchi et al. 1982)	UAG readthrough (Frey et al. 1989). Reading frame maintenance defect (Urbonavicius et al. 2001)	**TOB: −15.8 (*P* = .002) CIP: −2 (*P* = .04)**
*gidA mnmG*	5-carboxymethylamino-methyluridine-tRNA synthase. (m^5^U34)	Pleiotropic phenotypes on DNA replication, cell division, tmRNA tagging defect, reviewed in Nedialkova and Leidel (2015)	Decoding NNG codons (Kurata et al. 2008). Reading frame maintenance defect (Urbonavicius et al. 2001)	TOB: +1.9 (NS) **CIP: −2.3 (*P* = .05)**
*dusA*	tRNA-dihydrouridine synthase A	N/A (Bou-Nader et al. 2018)		**TOB : +3.8 (*P* = .0002)** CIP : +1.4 (NS)
*dusB*	tRNA-dihydrouridine synthase B	N/A (Bou-Nader et al. 2018)		**TOB : +12.2 (*P* = 10^−^^4^)** CIP : +1.3 (NS)
*miaA* N/A	tRNA dimethylallyltransferase (i6A37)	Mutator phenotype (Zhao et al. 2001). Reduced tetracycline resistance (Taylor et al. 1998). Evolvability of resistance to CIP (Mehi et al. 2013). Stress specific phenotype: RpoS/IraP translation (Aubee et al. 2016)	Reading frame maintenance defect (Urbonavicius et al. 2001)	**TOB: −18 (*P* = 10^−^^4^)**
*miaB*	Isopentenyl-adenosine A37 tRNA methylthiolase (ms2i6A37) U- starting codons	Stress specific phenotype: fur translation and iron levels (Vecerek et al. 2007)	Taylor et al. (1998)	**TOB : −5.9 (*P* = .0007)** CIP: +1.1 (NS)
*truA hisT*	tRNA pseudouridine38–40 synthase	Growth defect in minimal medium (Tsui et al. 1991)	Reading frame maintenance defect (Urbonavicius et al. 2001). Mistranslation (Bruni et al. 1977, Parker 1982)	TOB: +2.4 **(NS)** CIP: −2.1 **(NS)**
*truB*	tRNA pseudouridine55 synthase	Sensitivity to heatshock (Kinghorn et al. 2002)		TOB: −1.4 (NS) CIP: −1.9 (NS)
*truC*	tRNA pseudouridine65 synthase tRNAIle1 and tRNAAsp	N/A (Del Campo et al. 2001)		TOB: 1 CIP: 1
*trmA*	tRNA m^5^U54 methyltransferase and Y341 of tmRNA	N/A (Bjork and Neidhardt 1975). Sensitivity to heatshock of *truB trmA* (Kinghorn et al. 2002).		**TOB: −2.3 (*P* = .003)** CIP: −1.2 (NS)
*trmB*	tRNA m^7^G46 methyltransferase	N/A (De Bie et al. 2003)		**TOB: +2.7 (*P* = .008)** CIP: +3.4 (*p* = 0.06 NS)
*trmEmnmE*	5-carboxymethylaminomethyluridine-tRNA synthase m^5^U modification of U34 in tRNA	Pleiotropic, acts with *gidA*, see *gidA* above	Reading frame maintenance defect (Urbonavicius et al. 2001). UAG readthrough (Elseviers et al. 1984). Mistranslation (Hagervall et al. 1998)	**TOB: +3.9 (*P* = .01)** CIP: +2.4 (NS)
*trmH*	tRNA (Gm18) 2'-O-methyltransferase	N/A (Persson et al. 1997)		**TOB : +5.4 (*P* = .0002)** CIP : −1.3 (NS)
*trmK*	tRNA (m^1^A22)methyltransferase	Kimura et al. (2020)		TOB : 1 CIP : −1.3 (NS)
**rRNA modification (positions described in *Escherichia coli*)**				
*rlmB*	23S rRNA 2'-O-ribose G2251 methyltransferase	No obvious growth defect (Lovgren and Wikstrom 2001)		**TOB: −2.6 (*P* = .001) CIP: +2 (*P* = .05)**
*rlmI*	23S rRNA m5C1962 methyltransferase	Decrease in biofilm formation (Herzberg et al. 2006). Slight growth defect (Purta et al. 2008b).		TOB: −1.4 (NS) CIP: +1.3 (NS)
*rlmH* N/A	23S rRNA m3Ψ1915 methyltransferase	Slight growth defect (Purta et al. 2008a)		**TOB: −3 (*P* = .003)** CIP: −1.3 (NS)
*rlmE/rrmJ* N/A	23S rRNA 2'-O-ribose U2552 methyltransferase	Decreased growth rate (Caldas et al. 2000, Toh and Mankin 2008, Pletnev et al. 2020). Sparsomycin and tiamulin sensitive (Toh and Mankin 2008). Lincomycin sentitive (Caldas et al. 2000).	Frameshift and stop codon readthrough (Widerak et al. 2005). Accumulation of ribosomal subunit intermediates (Pletnev et al. 2020)	TOB: +1.2 (NS) **CIP: +825 (*P* = .003)**
*rsmB*	16S rRNA m^5^C967 methyltransferase	No obvious growth defect (Gu et al. 1999, Pletnev et al. 2020)	Accumulation of 17S rRNA (Pletnev et al. 2020). Translation initiation (Burakovsky et al. 2012, Arora et al. 2013)	**TOB: −6.7 (*P* = .007)** CIP: −2.9 NS
*rsmC*	16S rRNA m^2^G1207 methyltransferase	No obvious growth defect (Pletnev et al. 2020)	Correct folding of 16S rRNA (Gc et al. 2020)	**TOB: +2.3 (*P* = .05)** CIP: −1.2 (NS)
*rsmD*	16S rRNA m^2^G966 methyltransferase	No obvious growth defect (Lesnyak et al. 2007, Pletnev et al. 2020)	Translation initiation (Burakovsky et al. 2012, Arora et al. 2013).	**TOB: −5.7 (*P* = .006)** CIP: 1.1 (NS)
*rsmF/yebU*	16S rRNA m^5^C1407 methyltransferase	No obvious (Pletnev et al. 2020) or slight (Andersen and Douthwaite 2006) growth defect Increased resistance to some AGs reported (Gutierrez et al. 2012)	Role in translation initiation (Das et al. 2008)	**TOB: −2.5 (*P* = .002)** CIP: −1.8 (NS)
*rsmG* N/A	16S rRNA m7G527 methyltransferase	Mutations found in streptomycin resistant MTB clinical isolates (Okamoto et al. 2007)		**TOB: −7.7 (*P* = .0009)**
*rsmH* N/A	16S rRNA m^4^C1402 methyltransferase	No obvious growth defect (Dassain et al. 1999) *∆rsmH ∆rsmI* has a growth defect (Kimura and Suzuki 2010)	Decoding fidelity (Kimura and Suzuki 2010)	**TOB: −8.4 (*P* = .0009)**
*rsmI* N/A	16S rRNA 2'-O-ribose C1402 methyltransferase	No obvious growth defect *∆rsmH ∆rsmI* has a growth defect (Kimura and Suzuki 2010)	Decoding fidelity (Kimura and Suzuki 2010)	**TOB: −8.4 (*P* = 10^−^^4^)**
*rsuA*	16S rRNA pseudouridine516 synthase	No obvious growth defect (Conrad et al. 1999) Overexpression leads to resistance to HOCl (Chen et al. 2021)	Accumulation of 17S rRNA (the present study)	**TOB: −2.3 (*P* = .01)** CIP: 1
*rluB*	23S rRNA pseudouridine2605 synthase	No obvious growth defect (Del Campo et al. 2001, Toh and Mankin 2008). Increased CM and linezolid sensitivity (Toh and Mankin 2008)	50S subunit maturation (Jiang et al. 2007)	**TOB: +9.4 (*P* = 10^−^^4^)** CIP: +1.2 (NS)
*rluC* N/A	23S rRNA pseudouridine955/2504/2580 synthase	No obvious growth defect (Conrad et al. 1998). Cold sensitivity (Jiang et al. 2007) clindamycin, linezolid, and tiamulin sensitivity (Toh and Mankin 2008)		**TOB: +1.8 (*P* = 10^−^^4^)** CIP: +1.3 (NS)
*rluD*	23S rRNA pseudouridine1911/1915/1917 synthase	Reported to cause a large growth defect in *Escherichia coli* but independently of pseudourdines (Gutgsell et al. 2001)	Ribosome assembly (Gutgsell et al. 2005)	**TOB: +1.4 (*P* = .03) CIP: +6.6 (*P* = .002)**
*rluE*	23S rRNA pseudouridine2457 synthase	No obvious growth defect (Del Campo et al. 2001)		**TOB: −1.5 (*P* = .002)** CIP: −2.4 (NS)
**Modification of both tRNA and rRNA**				
*rluF*	23S rRNA pseudouridine2604/tRNATyr pseudouridine35 synthase	No obvious growth defect (Del Campo et al. 2001, Toh and Mankin 2008, Pletnev et al. 2020). Decreased linezolid resistance (Toh and Mankin 2008)	Effect on translation of Tyr codons (Addepalli and Limbach 2016)	TOB: −1.2 (NS) CIP: −1.2 (NS)
*rlmN*	tRNA m2A37 methyltransferase/23S rRNA m2A2503 methyltransferase	No obvious growth defect (Benitez-Paez et al. 2012). Slightly increased susceptibility to certain peptidyl transferase-targeting antibiotics (Toh and Mankin 2008)	UAG readthrough (Benitez-Paez et al. 2012)	**TOB: +2.3 (*P* = .005) CIP: −2.2 (*P* = .0003)**

The most important TN-seq hits for TOB include: (i) tRNA modification genes for which inactivation is detrimental: incorporation of queuosine (Q) by *tgt* (together with the Q synthesis genes *queADEF)*, and i6A37/ms2i6A37 by *miaA/miaB*; or beneficial: dihydrouridine (D) incorporation by *dusB, dusA*; and methylation by *trmH, rlmN*; and m^5^U34 incorporation (*gidA*, also called *mnmG*); (ii) rRNA modifications for which inactivation is detrimental: methylation by *rsmI, rsmF, rsmG, rsmH, rsmB, rsmD*, and pseudouridine (ψ) incorporation by *rsuA*; or beneficial: ψ by *rluB*. Note that *rsmG* and *rsmF* mutants have already been associated with increased AG resistance (Table 1, and references therein), but our results suggest decreased fitness in AGs for these mutants.

For CIP: (i) tRNA modification genes for which inactivation is detrimental were responsible for ψ incorporation (*truA)* and methylation (*rlmN)*; (ii) rRNA methylation genes were also identified in CIP, some at different positions than those in TOB (detrimental inactivation of *rsmB* and beneficial *rlmE*). Note that RlmN can modify both tRNA and rRNA.

Overall, several nontrivial observations stem from our results: first, the effect of inactivation of these genes on fitness can either be negative (e.g. *tgt* in TOB), or positive (e.g. *dusB* in TOB). Second, their impact seems to be an antibiotic specific one. For instance, the inactivation of *dusB*/*tgt*/*rluB* strongly impacts the fitness in TOB, but not in CIP. Our previous transcriptomics results suggest that sub-MIC TOB could induce the stringent response (Carvalho et al. 2021a). Since RNA modifications could affect ribosome function, we asked whether the absence of certain modifications could induce the stringent response and whether there is a correlation between stringent response induction and changes in fitness upon antibiotic treatment. We constructed a *gfp* fusion under the control of the rRNA *P1rrn* promoter, which is down-regulated upon stringent response induction (Kolmsee et al. 2011) and followed fluorescence at various time points throughout growth (Figure S1, Supporting Information). We found that the stringent response is significantly induced by sub-MIC TOB [i.e. decrease of fluorescence; Figure S1 (Supporting Information), last graph], but no induction was observed for RNA modification deletion mutants. Unexpectedly, slightly increased fluorescence was observed during early exponential phase for *∆gidA/∆trmE*, as well as *∆truA, ∆rsuA, ∆rsmB*, and *∆rluD*, showing increased transcription from the P1*rrnB* promoter (e.g. possibly through Fis or another transcriptional activator).

Altogether, these observations suggest that the loss of a given modification may affect the bacterial response in a specific way rather than through a general effect of all modifications on translation. While AGs, which target the ribosome, could be expected to impact translation related genes, it was surprising that the response to CIP, which targets DNA, also involves several RNA modification genes, suggesting that the involvement of RNA modifications may be fundamental upon stress due to antibiotics from different families.

### RNA modification gene deletions impact fitness during growth in sub-MIC antibiotics

We next constructed *V. cholerae* deletion mutants for 23 of the identified RNA modification genes, selected in TN-seq data for having no (or slight) effect on fitness during growth in the absence of antibiotics. Many have no known physiological defect, and were not previously associated to antibiotic related phenotypes (Table 1). The following genes were excluded from further study either for known effects on growth: *miaA, rsmA*, and *rlmE;* or for known AG related phenotypes: *rsmG, rsmA*, and *rsmH* (Zou et al. 2018). We chose *trmK* as a neutral control for TOB and CIP, as it showed no variation in our TN-seq screens.

**Figure 3:**
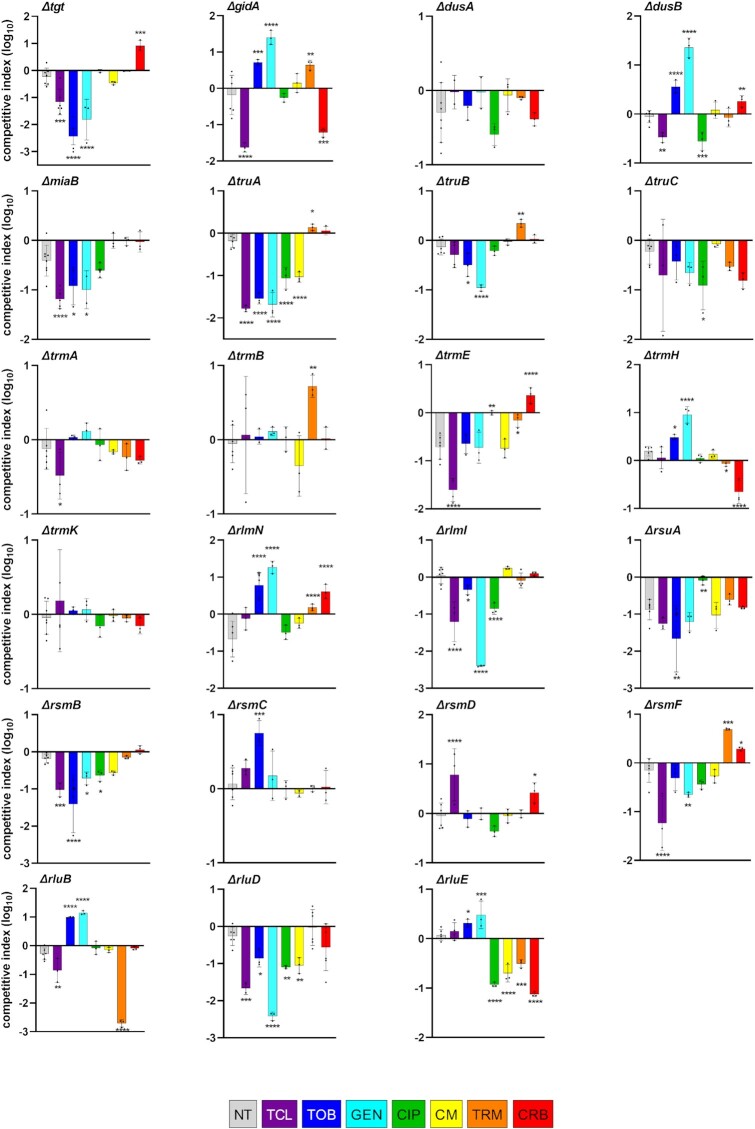
Impact of RNA modification gene deletions on fitness during growth in sub-MIC antibiotics. *In vitro* competition experiments of *V. cholerae* WT and mutant strains in the absence or presence of different antibiotics at sub-MICs (50% of the MIC). TCL: triclosan 0.01 mM; TOB: tobramycin 0.6 μg/ml; GEN: gentamicin 0.5 μg/ml; CIP: ciprofloxacin 0.002 µg/ml, CM: chloramphenicol 0.4 µg/ml, TRM: trimethoprim 0.4 µg/ml, and CRB: carbenicillin 2.5 µg/ml. The *y*-axis represents log_10_ of competitive index value calculated as described in the methods. A competitive index of 1 indicates equal growth of both strains. NT: no antibiotic treatment. For multiple comparisons, we used one-way ANOVA on GraphPad Prism. **** means *P*< .0001, *** means *P*< .001, ** means *P*< .01, and * means *P*< .05. Only significant *P*-values are represented. Number of replicates for each experiment: 3 < *n* < 8.

Since growth curves of monocultures of the mutants were similar to that of the wild type (WT) in the absence of treatment (not shown), we decided to perform competition experiments between mutants and the WT strain, to assess effects on fitness in sub-MICs of six different antibiotics: the AGs TOB and gentamicin (GEN), the fluoroquinolone CIP, as used in our TN-seq screen, and additionally the β-lactam carbenicillin (CRB) targeting the cell envelope, chloramphenicol (CM) targeting translation elongation and trimethoprim (TRM), which inhibits thymidine synthesis interfering with DNA synthesis. Figure 3 shows the competitive index of mutants compared to WT. The results are summarized in Table 2.

**Table 2.** Summary of the competitive index of the *V. cholerae* deletion mutants compared to WT during growth in sub-MIC antibiotics.

*Gene*	TN-seq	TOB	GEN	Ec	TN-seq	CIP	CM	TRM	CRB	TCL
	TOB	fitness	fitness	TOB	CIP	fitness	fitness	fitness	fitness	fitness
*tgt*	**-**	**-**	**-**	+/-	**-**				**+**	**-**
*gidA/mnmG*	(+)	+	+	-	**-**			+	-	-
*dusA*	**+**			+						
*dusB*	**+**	**+**	**+**	+		**-**			**+**	**-**
*miaB*	**-**	**-**	**-**	N/A		**-**				**-**
*truA*	(+)	-	-	N/A	(-)	-	-			-
*truB*	(-)	-	-	N/A	(-)			+		
*truC*				N/A		-				
*trmA*	**-**			N/A						**-**
*trmB*	**+**			N/A	(+)			**+**		
*trmE/mnmE*	**+**			-	(+)	**+**			**+**	**-**
*trmH*	**+**	**+**	**+**	N/A					**-**	
*trmK*				N/A						
*rlmI*	(-)	-	-	N/A		-				-
*rsmB*	**-**	**-**	**-**	N/A	(-)	**-**				**-**
*rsmC*	**+**	**+**	**+**	+						
*rsmD*	**-**			-					**+**	**+**
*rsmF*	**-**	**-**	**-**	N/A	(-)			**+**	**+**	**-**
*rsuA*	**-**	**-**	**-**	+		**+**				
*rluB*	**+**	**+**	**+**	+	(+)			**-**		**-**
*rluD*	**+**	**-**	**-**	N/A	**+**	**-**	**-**			**-**
*rluE*	**-**	**+**	**+**	N/A	(-)	**-**	**-**	**-**	**-**	
*rlmN*	**+**	**+**	**+**	N/A	**-**			**+**	**+**	

**Figure 4:**
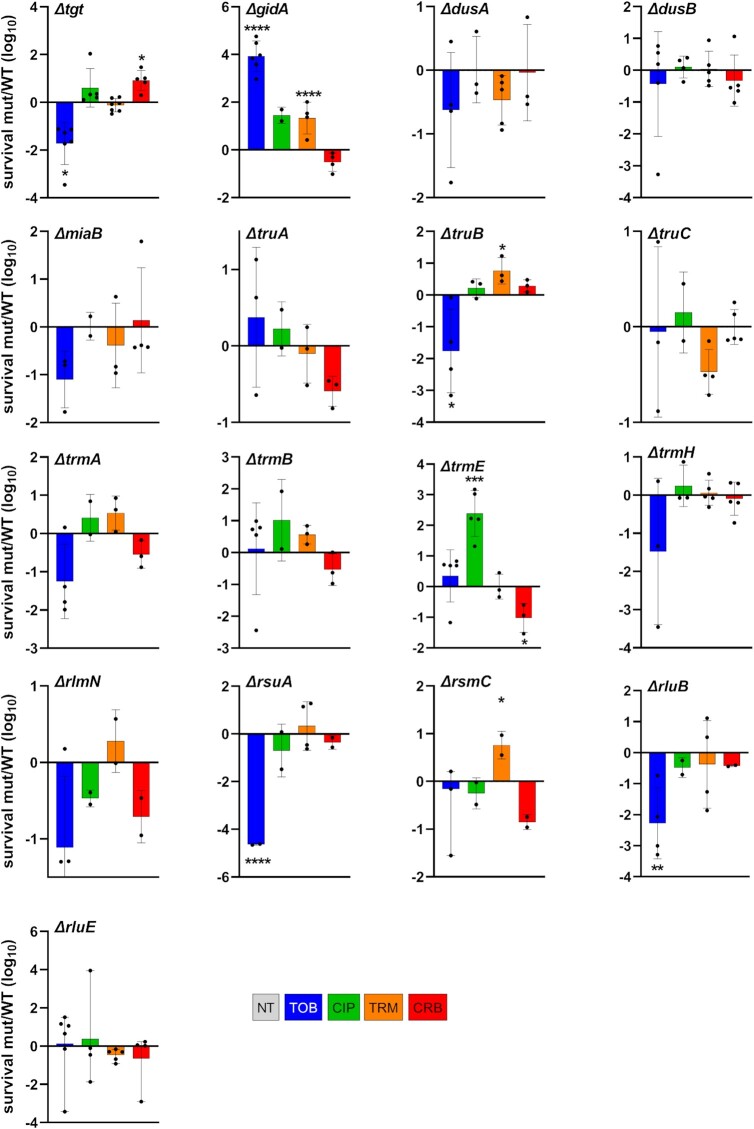
Survival to lethal antibiotic treatment. *Vibrio cholerae* WT and deletion mutant cultures were grown without antibiotics up to early exponential phase. Total number of bacteria was determined by plating on MH plates before addition of the indicated antibiotic at >MIC, at time T0. After 20 h of incubation with the antibiotic, the number of surviving bacteria was determined by plating on MH plates (T20). Survival was calculated for each strain by dividing the number of surviving bacteria at T20 by the initial number of bacteria. The *y*-axis represents the log10 survival ratio of a given mutant over the survival of the WT strain. Antibiotic concentrations: TOB 10 µg/ml, CIP 0.04 µg/ml, TRM 50 µg/ml, and CRB 50 µg/ml. Means and geometric means for logarithmic values were calculated using GraphPad Prism. For multiple comparisons, we used one-way ANOVA on GraphPad Prism. **** means *P*< .0001, *** means *P*< .001, ** means *P*< .01, and * means *P* < .05. Only significant *P*-values are represented. Number of replicates for each experiment: 3 < n < 8.

“-” means that deletion of the gene decreases fitness (> 2-fold change). “+” means deletion of the gene increases fitness (> 2-fold change). Parentheses indicate that the *P*-value was not significant. An empty cell means that no significant effect of deletion was observed. “Ec TOB:” column summarizing results from *Escherichia coli* growth curves in sub-MIC TOB (Fig. 4), “N/A” means that the corresponding mutant was not tested in *E. coli*.

As expected, deletions of the majority of tested genes (with the exception of *trmE, rsuA*, and *rlmN*) have no or little effect on competitive index during growth in the absence of antibiotics (Fig. 3), emphasizing their specific role during stress, here sub-MIC antibiotics.

For the AGs TOB and GEN, among tested genes, deletion of *tgt, miaB, truA, truB, rlmI, rsmB, rsmF*, and*rluD* decreased fitness, while deletion of *gidA, dusB, trmH, rlmN, rsmC, rluB*, and*rluE* conferred a growth advantage (Fig. 3). These results were consistent with TN-seq data, with the exception of *truA, truB, rluE*, and*rluD* for which the TN-seq data were not statistically significant, and *dusA* and *trmB* for which the growth advantage observed in TN-seq is not found in competition experiments. For CIP, deletions of *dusB, miaB, truA, truC, rlmI, rsmB, rluD*, and*rluE* were disadvantageous, whereas ∆*trmE* and ∆*rsuA* strains appear to lose the fitness disadvantage they show in absence of CIP compared to WT. Once again, results were consistent with the statistically significant TN-seq results, except for the *rluD* gene. For CM, *truA, rluD*, and *rluE* deletions were detrimental. For TRM, *rluB* and *rluE* deletions were detrimental, while deletions of *gidA, truB, trmB, rlmN*, and*rsmF* conferred a low (up to 10x) but statistically significant growth advantage. For CRB, detrimental deletions were *gidA, trmH*, and*rluE*, and advantageous deletions were *tgt, dusB, trmE, rlmN, rsmD*, and*rsmF*.

In order to test whether these modification genes could be important for the response to another type of stress, we also performed competitions in the presence of the biocide triclosan (TCL), at 50% of the MIC. TCL inhibits fatty acid synthesis and can be found in antiseptic consumer products. It has been a subject of concern for its impact on the aquatic environment (Dhillon et al. 2015) and antibiotic resistance development (Wesgate et al. 2016). Again, while deletion of many RNA modification genes decreased fitness in TCL (*tgt, gidA, dusB, miaB, truA, trmA, trmE, rlmI, rsmB, rsmF, rluB*, and *rluD*), some were neutral (*dusA, trmB, trmH, rlmN, rluE*, and*trmK*), and one was beneficial (*rsmD*).

These results globally confirm that the effect of a given modification gene is not a general one on viability but an antibiotic specific one. For instance, regarding tRNA modifications, upon AG treatment (TOB and GEN), deletion of *tgt* confers a clear 10–1000x disadvantage, while it has no major effect in CIP, TRM, and CM and appears to be 10x advantageous in CRB. Deletion of *truA* confers a up to 100x fitness defect in AGs, CIP, and CM, but is neutral in TRM and CRB. Deletion of *truB* also appears to affect specifically growth in AGs. Deletions of *dusB/rlmN*, and *gidA*/*trmH* are highly (10–100x) beneficial in AGs, but respectively deleterious or neutral in CIP. *rlmN* deletion also confers a slight advantage in TRM and CRB. Deletion of *trmA* shows no major effect in any antibiotics, while *trmB* deletion is only beneficial in TRM. Regarding rRNA modifications, *rluB* shows a striking phenotype with 10x beneficial deletion in AGs, highly (1000x) deleterious in TRM, and neutral in presence of the other antibiotics (Fig. 2). Of note, *gidA* (*mnmG)/trmE (mnmE)* are known to have pleiotropic phenotypes due to effects on translation (Bregeon et al. 2001), chromosome replication, and cell division (Alam and Clark 1991, Ogawa and Okazaki 1991, Theisen et al. 1993, Lies et al. 2015, de Crecy-Lagard and Jaroch 2021), in addition to effects on tRNA modification (Elseviers et al. 1984). Regarding TCL, many RNA modification gene deletions confer a fitness defect. However, the fact that deletion of *rsmD* is beneficial indicates that the absence or the decrease of the levels of a given RNA modification can also allow a better fitness upon exposure to toxic chemicals such as anti-septics.

### RNA modification gene deletions impact tolerance to high doses of antibiotics without changing the resistance

Next, we addressed whether these genes could be involved in the response to lethal antibiotic concentrations. We first determined the MIC of TOB, CIP, TRM, and CRB for each deletion mutant (Table 3). Slight decreases in the MIC of TOB (x 0.9) was observed for *∆rlmI* and *∆rsmD*. Slight increases in MIC were observed for *∆gidA* and *∆rluB* in TOB (x1.6), for *∆gidA, ∆rluD*, and*∆trmE* in CIP (x1.2), (x1.1) for *∆rlmN* in AMP (as a substitute for CRB) and for *∆trmE* and *∆truC* in TRM (x1.6). Besides these small changes, we found no major differences in MICs, consistent with the fact that these genes were not previously associated with antibiotic resistance phenotypes.

**Table 3.** MICs determined using Etests.

*Vibrio cholerae*	TOB	CIP	AMP	TRM
*WT*	0.75–1.2	0.0020–0.0030	4 +/−1	0.4 +/− 0.1
*tgt*	0.75–1	0.0020	4	0.58
*dusA*	1–1	0.0020–0.0030	4	0.5
*dusB*	1–1	0.0020–0.0030	4	0.48
*gidA*	2–2*	0.0039*	4	0.37
*trmA*	0.9–1	0.0020	3.9	0.37
*trmB*	1–1.2	0.0020	4.3	0.48
*miaB*	0.9–1	0.0020	4	0.38
*rsmF*	1	0.0020	4.5	0.4
*rlmI*	1	0.0020	4.2	0.4
*truA*	0.8	0.0020	3.5	0.39
*rluD*	0.75	0.0036*	4	0.5
*trmE*	0.75–1.2	0.0036*	5	0.8*
*trmK*	1–1.2	0.0020	4	0.38
*rsmB*	1	0.0020	4.1	0.48
*truB*	1–1.2	0.0020	4	0.47
*rluF*	1–1.2	0.0020	4	0.43
*truC*	0.75	0.0035	5	0.8*
*trmH*	1	0.0025	4.3	0.5
*rlmN*	1	0.0025	5.5*	0.38
*rlmI*	0.7**	0.0028	4.5	0.44
*rsuA*	0.75	0.0030	3.5	0.4
*rsmC*	1.5	0.0028	4.8	0.38
*rsmD*	0.7**	0.0028	3.5	0.38
*rsmF*	0.8	0.0032	3.8	0.38
*rluB*	1.5*	0.0030	4	0.5
*rluE*	0.75	0.0028	5	0.5

Green: increase MIC. Blue: decrease. TOB: tobramycin. CIP: ciprofloxacin. AMP: ampicillin (used for CRB). TRM: trimethoprim. * shows increase. ** shows decrease.

We then tested the survival to lethal concentrations of antibiotic, i.e. the tolerance (Fig. 4): WT and mutant bacteria were grown to early exponential phase and then treated for 20 h with 10xMIC of TOB, CIP, TRM, and CRB as previously performed (Lang et al. 2021).

For 10 mutants out of 17 tested (among which nine tRNA modification mutants), survival profiles were consistent with fitness profiles shown in Fig. 3. These were mutants *tgt, gidA, truB, trmE* (except in CRB), and *rsuA*, for which increased fitness corresponded to increased tolerance and vice-versa; and *dusA, miaB, truC, trmA*, and*trmB* for which the absence of statistically significant effect on tolerance was also consistent with the absence of differences in fitness. This suggest that a fitness (dis)advantage in sub-MIC antibiotics in the absence of tRNA (and rRNA) modifications may also impact tolerance to lethal doses of the same antibiotic, without changing the MIC.

For three mutants, *dusB, trmH*, and *rluE*, no significant effect on tolerance was generally observed at 20 h of lethal treatment, while deletion of these genes positively affected fitness in sub-MIC TOB. In order to address whether differences in tolerance could be detected at earlier times of treatment, we repeated the experiments and spotted cultures after 30 min, 1, and 2 h of antibiotic treatment instead of 20 h (Figure S2, Supporting Information). While *∆dusB* tolerance was still similar to that of WT, *∆trmH* and *∆rluE* strains displayed increased tolerance to TOB at 30 min and 1 h of treatment, consistent with their beneficial effect on fitness in sub-MIC TOB.

For the remaining four mutants, among which three rRNA modification mutants, we observed contradictory phenotypes between fitness and tolerance at 20 h, i.e. decreased TOB tolerance at 20 h in beneficial deletion mutants *rlmN, rsmC*, and*rluB*; and CRB for *trmE*. However, at earlier time point as described above, and consistent with fitness profiles, TOB tolerance is clearly increased in *rlmN, rsmC*, and*rluB* (Figure S2, Supporting Information), suggesting that mutants with fitness advantage in sub-MIC TOB also survive longer in the presence of lethal TOB concentrations. However, the final survival after 20 h of treatment is not increased, consistent with unchanged MICs. This phenotype is a characteristic of antibiotic tolerant populations (Balaban et al. 2019). CRB tolerance of ∆*trmE* remains lower than WT (not shown).

**Figure 5:**
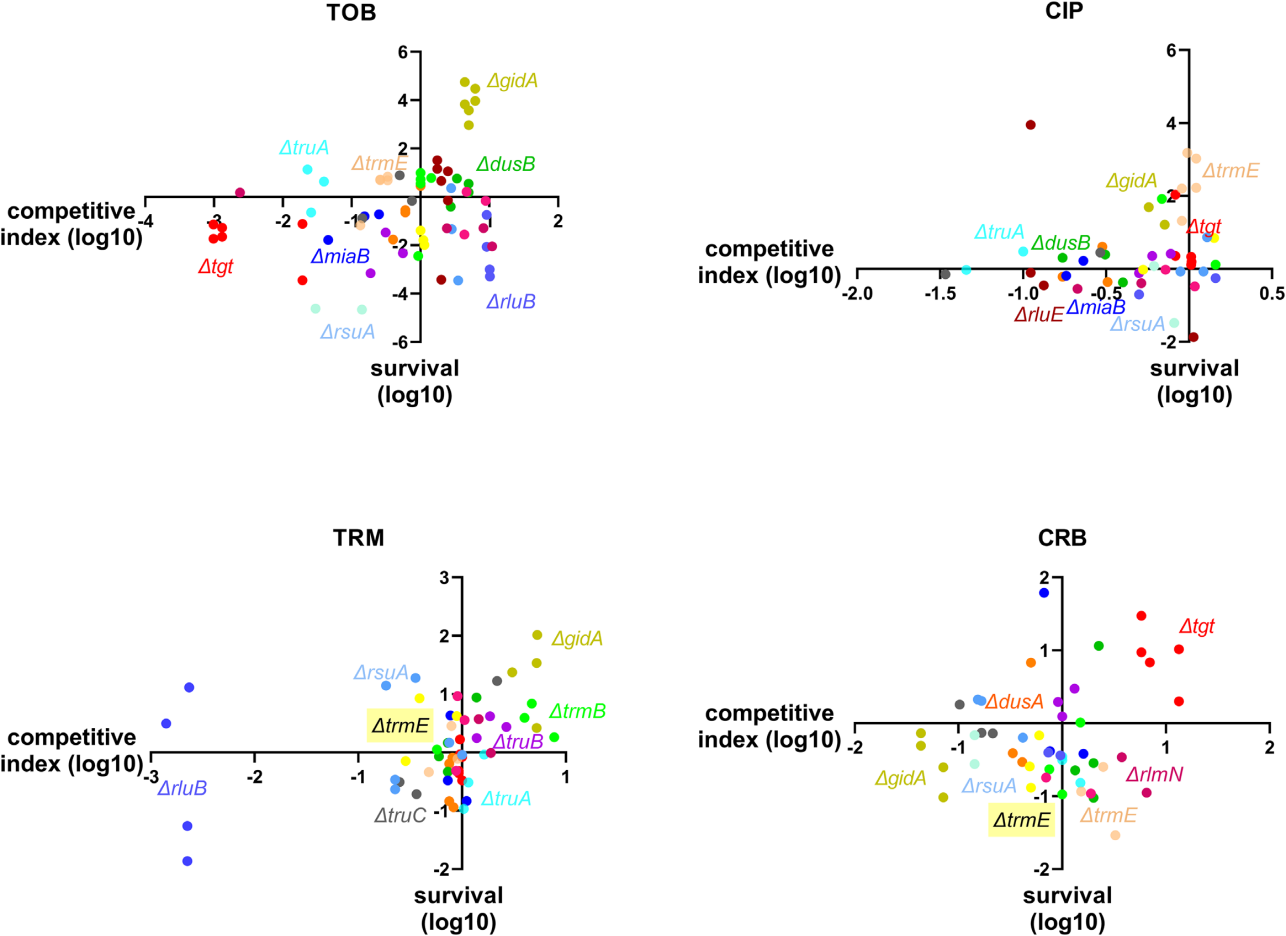
Fitness versus survival. For each antibiotic, survival values from Fig. 3 (*y*-axis, log10 values) were plotted against competitive index values from Fig. 2 (*x*-axis, log10 values). TOB: tobramycin, CIP: ciprofloxacin, TRM: trimethoprim, and CRB: carbenicillin. Names of genes with significant changes are indicated.

To evaluate the levels of correlation between competitive index and survival phenotypes, we plotted the two sets of data against each other, and for each antibiotic (Fig. 5). We observe that there is, or is not, a correlation between growth in sub-MIC and survival, depending on the gene deletion and the antibiotics. *∆tgt, ∆gidA, ∆dusB, ∆rsuA*, and*∆miaB* for TOB; *∆gidA* and*∆trmB* for TRM; and *∆tgt, ∆gidA*, and*∆trmE* for CRB, show similar tendencies for growth in sub-MIC and survival to 20 h lethal antibiotic treatment, meaning that in these cases, better growth in sub-MIC and better survival may occur through the same mechanisms. In other examples, the two phenotypes show opposite directions, as for *∆rluB* in TOB or *∆rlmN* in CRB. Interesting to note, for CIP and TRM, which target DNA and replication, the two phenotypes seem to be uncorrelated in most cases, while in TOB and CRB both phenotypes may vary for a given mutant.

Overall, tolerance profiles of several mutants correlate with their fitness profiles in sub-MICs of antibiotics. For those, such as *∆dusB*, with increase in fitness but not in tolerance, the mechanisms remain to be determined, and their phenotypes suggest that the effects of RNA modifications during growth in stressful conditions (sub-MIC antibiotic) do not necessarily affect survival to high antibiotic doses. rRNA modifications in particular could be expected to have structural effects on ribosomes, which could lead to pleiotropic effects, and could potentially explain this discrepancy.

One such effect is 17S rRNA accumulation, due to a defect of maturation to 16S rRNA (previously shown for *∆rsmA* (Smith et al. 2018) and *∆rsmB*, Table 1). We visualized rRNA species purified form exponentially growing WT and RNA modification deletion mutants (Figure S3, Supporting Information). We find accumulation of a pre-16S, consistent with 17S, rRNA species for *∆rsuA*, for which fitness is most affected also in the absence of antibiotics. RsuA is a 16S rRNA pseudouridine synthase. Apart from *∆rsuA*, we observed no differences in rRNA species between the other tested deletion mutants and the WT. This is consistent with the fact that these strains do not exhibit any major growth defect in the absence of antibiotics. Further study is needed to clarify the role of identified rRNA modifications in antibiotic specific survival.

**Figure 6:**
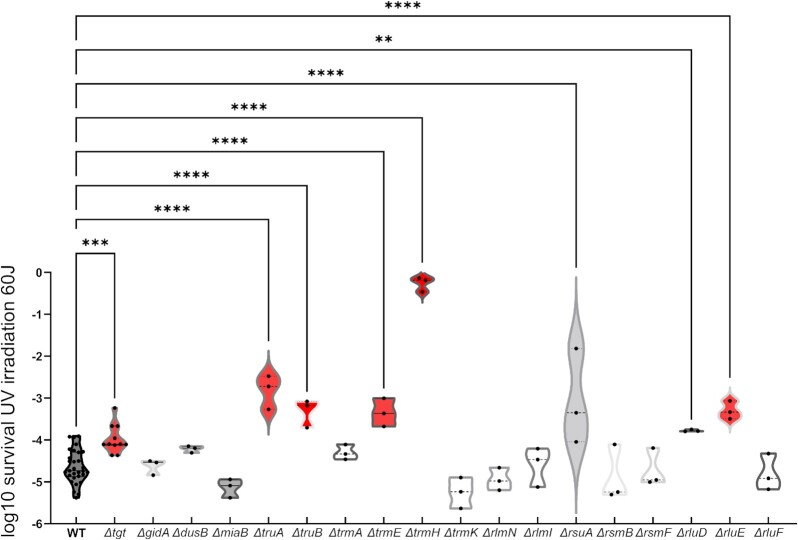
Survival of *V. cholerae* WT and RNA modification deletion mutants after UV irradiation. Survival of indicated *∆-mutant* relative to WT after UV irradiation at 60 Joules/m^2^. For multiple comparisons, we used one-way ANOVA on GraphPad Prism. **** means *P*< .0001, *** means *P*< .001, and ** means *P*< .01.

We also evaluated whether deletion of these RNA modification genes could have any effect on DNA mutation rates by quantifying the appearance of spontaneous rifampicin resistant mutants (Figure S4, Supporting Information), and found no major effect on mutation rates except for *∆gidA*. This confirms that the fitness advantage/disadvantage conferred by RNA modification gene deletions are not due to an effect on mutation rates and/or accumulation of mutations. Finally, we asked whether RNA modification gene deletions could also have a specific impact on stresses other than antibiotic or chemical treatments. One such stress is UV irradiation. While 10 mutants did not show any difference with the WT strain, survival after UV irradiation was increased in seven mutants (*tgt, truA, truB, trmE, trmH, rluD*, and *rluE*; Fig. 6), suggesting that RNA modifications impact the response to different stress conditions.

### RNA modification gene deletions also impact *Escherichia coli* growth in sub-MIC antibiotics

**Figure 7:**
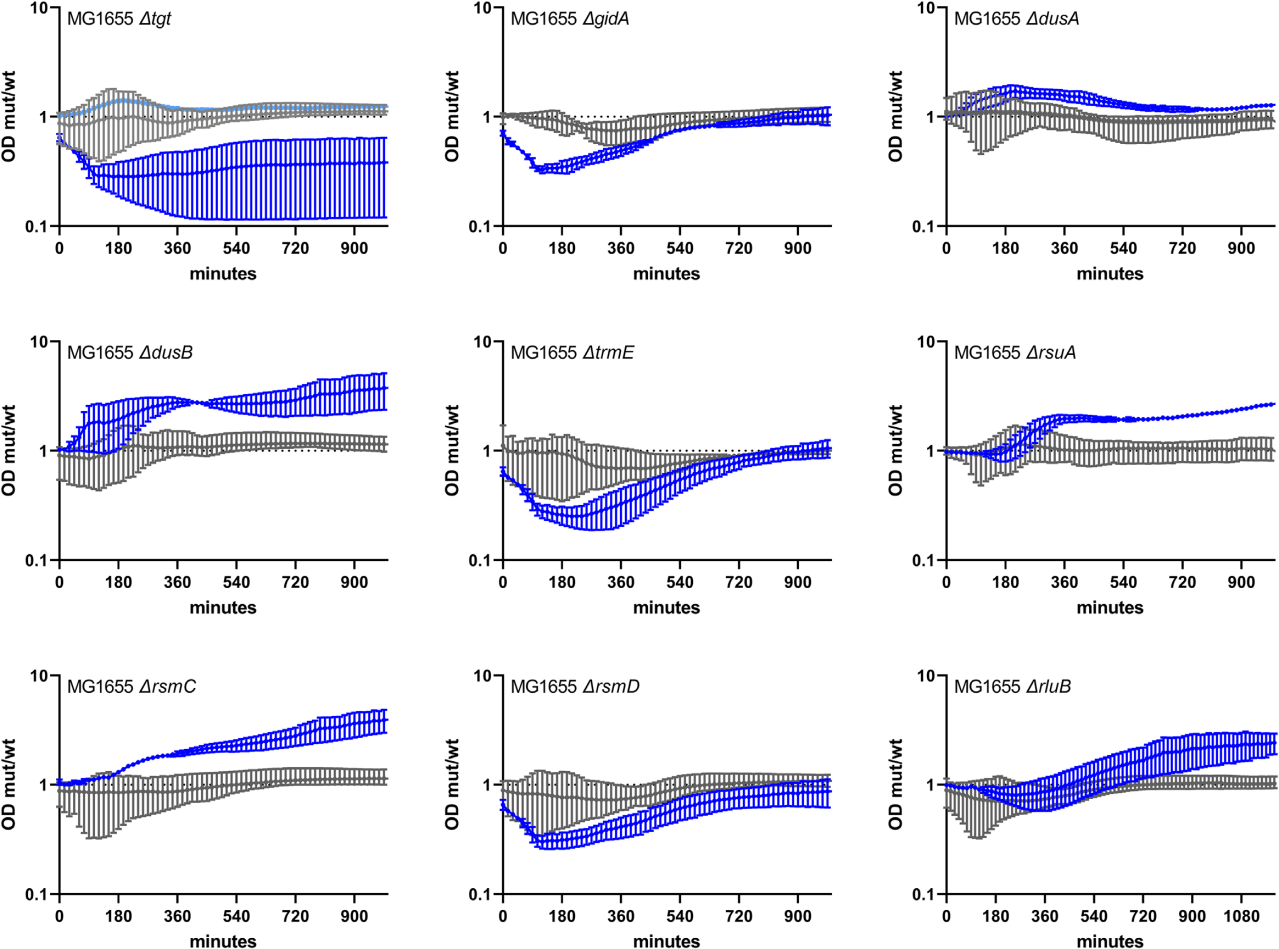
Growth of *E. coli* MG1655 WT and derivatives deleted for selected RNA modification genes in sub-MIC TOB. Overnight cultures were diluted 100x in fresh MH medium, on 96-well plates. Plates were incubated with shaking in TECAN plate reader device at 37°C, OD 620 nm was measured every 15 min. Grey: no treatment. Blue: sub-MIC TOB, at 0.2 µg/ml (50% of the MIC for MG1655 in MH liquid culture). The *y*-axis represents the OD of the mutant divided by the OD of the WT strain in the same growth conditions, and thus reflects slower (below 1) or faster (above 1) growth. Standard deviation is shown.

We next sought to determine whether RNA modification genes also play similar roles in bacterial species other than *V. cholerae*. We constructed deletion mutants in *Escherichia coli* MG1655 of nine genes selected for their positive (*gidA, dusB, rsmC*, and *rluB*), neutral (*dusA* and *rsmD*), and negative (*tgt, trmE*, and *rsuA*) impact on *V. cholerae* fitness in sub-MIC TOB (Fig. 7; Figure S5, Supporting Information). Note that inactivation of *dusA* and *rsmD* were observed to be respectively beneficial and deleterious in *V. cholerae* TN-seq data, but not in competitions. Growth curves in 50% MIC TOB display similar and dissimilar phenotypes in *E. coli* compared to those observed for *V. cholerae*. First, similar to *V. cholerae*, (i) deletions of *dusB, rsmC, rluB*, and *dusA* and (ii) deletions of *tgt, trmE*, and *rsmD*, have a positive and a negative impact, respectively, on growth in sub-MIC TOB in *E. coli*. For ∆*tgt*, we also had some replicates with no observable effect in sub-MIC TOB (curve in light blue), suggesting heterogeneous response to TOB stress in this mutant in *E. coli*. On the other hand, unlike in *V. cholerae*, ∆*gidA* decreases while ∆*rsuA* improves growth of *E. coli* MG1655 in TOB. Note that synteny is conserved between the two organisms for these genes, hence the differences cannot be attributed to an effect of the deletions on surrounding genes. We also observed the same growth profiles (Figure S6, Supporting Information) in an *E. coli* BW25113 (Keio) strain, for all mutants except for the BW25113 ∆*tgt* strain, which unexpectedly has a positive impact on growth in sub-MIC TOB in this genetic context. Note that growth curves show differences in growth but not necessarily in fitness as it is the case for competition experiments where WT and mutant cultures are mixed. Nonetheless, these results show that the involvement of RNA modification genes in the response to sub-MIC antibiotic stress is not specific to *V. cholerae* and can be extended to other bacterial species, although their antibiotic related effects may sometimes be species and even strain-specific.

## Discussion

Using antibiotics at sub-MICs, we identify here the importance of rRNA and tRNA modification genes, not previously associated to any antibiotic resistance/tolerance phenotypes [Table S1 (Supporting Information), and references therein]. Among these are rRNA methylation factors RsmB/C/D/H/I and RlmI, rRNA pseudouridine synthases RsuA and RluD; and Tgt, DusB, TruA/B/C, TrmA/B/E/H, and RlmN for tRNA modifications.

Most t/rRNA modifications influence translation rate, fidelity and precision of codon decoding (Chan et al. 2018). Errors in decoding can lead to transient tolerance to stress (Samhita et al. 2020), increasing the cell’s chances to acquire genetic mutations allowing adaptation (Bratulic et al. 2017). For instance, increased survival after 20 h antibiotic treatment as described above for several mutants, may be due to tolerance or persistence, a transient state of phenotypic (nonheritable) resistance to lethal antibiotic concentrations. The idea that RNA modifications can act on such phenotypic adaptation is interesting, and worth pursuing.

Since the genetic code is degenerate, and one tRNA can decode several codons, decoding efficiency can logically be impacted by tRNA modification (Quax et al. 2015). Thus, the link between tRNA modification-dependent differences in translation, proteome diversity, and the bacterial response to antibiotics and more generally to changing environments, is an attractive area to explore further. It is known that codon usage has an impact on translation (Gingold and Pilpel 2011, Nieuwkoop et al. 2020, Krafczyk et al. 2021). Highly translated mRNAs, such as those of ribosomal proteins, display a codon usage profile different than the general codon usage in the genome (Plotkin and Kudla 2011). It was proposed that codon usage of highly expressed genes is determined by the abundance of tRNAs, so as to prevent titration of tRNAs, hence allowing efficient translation of the rest of the proteome (Frumkin et al. 2018). We can speculate that codon usage of these genes can also be a function of decoding efficiency displayed by modified versus unmodified tRNAs. Various transcriptional regulators also show codon usage biases, and RNA modifications may impact their translation, and thus lead to differential transcription of the regulon that they control (Vecerek et al. 2007, Aubee et al. 2016, Chionh et al. 2016, Thongdee et al. 2019).

Stress-regulated RNA modifications would facilitate homeostasis by reprogramming the translation of stress response genes (Pollo-Oliveira and de Crecy-Lagard 2019). Although RNA modifications were initially thought to be static, studies reveal the existence of dynamic modifications depending on growth phase and rate (Moukadiri et al. 2014), environmental changes [reviewed in Barraud and Tisne (2019), Georgeson and Schwartz (2021)], or stress (Galvanin et al. 2020), leading to differential translation of stress response transcripts and translational reprogramming (Pollo-Oliveira and de Crecy-Lagard 2019). In this process, RNA modifications and modification levels have an impact on the translation of regulators, which were thus defined as modification tunable transcripts, or MoTTs (Endres et al. 2015).

Such processes were described in *E. coli* for the general stress sigma factor *rpoS* carrying leucine codons necessitating MiaA-modified tRNAs (Aubee et al. 2016); for the iron sensing *fur* regulator, carrying serine codons decoded by MiaB-modified tRNA, in response to low iron (Vecerek et al. 2007); for the response to magnesium levels through TrmD modification dependent decoding of proline codons in *mgtA* (Hou et al. 2017). In *Pseudomonas aeruginosa*, TrmB modification increases translation efficiency of phenylalanine and aspartate enriched catalase mRNAs during oxidative stress (Thongdee et al. 2019), suggesting tRNA methylation mediated translational response to H_2_O_2_. During the mycobacterial response to hypoxic stress (Chionh et al. 2016), differential translation of specific stress response genes was linked, first *in silico*, then experimentally, to their codon usage bias. Our results highlight tRNA dependent translational reprogramming as a promising subject to be addressed in bacteria in regard to antibiotic stress.

This study reveals the existence of an epigenetic control of the response to sub-MIC antibiotics at the RNA level, adding upon our previous report of an epigenetic tolerance to AGs at DNA level (Carvalho et al. 2021b). Such a response may also involve gene sequences which coevolve with the specific bacterial species so that translational regulation of the response to antibiotics becomes associated with other stress response genes bearing differentially decoded sequences, i.e. MoTTs. It can also not be excluded that certain of these RNA modification enzymes also exert their effect through mRNA modification (Hurt et al. 2007, Eyler et al. 2019). Molecular study of codon decoding particularities of each RNA modification mutant, coupled to proteomics, ribosome profiling and *in silico* analysis of genes with differential codon usage, could allow for the identification of specific pathways post-transcriptionally regulated by a given RNA modification. A sequence specific action was recently shown for antibiotics targeting the ribosome (Syroegin et al. 2022, Tsai et al. 2022), where the identity of the amino-acid at the penultimate position determines whether the ribosome will stop or continue translation in the presence of the antibiotic. In this case, a specific 23S rRNA methylation (by Cfr) leads to structural changes, which hinders antibiotic access. Interestingly, two very similar antibiotics, which have the same sequence specificities (here linezolid and radezolid), can be affected differently by rRNA modifications, inhibiting one and not the other. Such results, together with our observation that multiple rRNA modifications can impact antibiotic related phenotypes, may initiate future studies raising the possibility of the development of antibiotics inhibiting translation of specific proteins.

## Mat and Met

### Bacterial strains and plasmids

All *V. cholerae* strains used in this study are derivative of *V. cholerae* N16961 *hapR*+, and were constructed by allelic exchange. *Vibrio cholerae* mutants were constructed in the ∆*lacZ* strain (K329). All *E. coli* strains used in this work are derivatives of *E. coli* MG1655, and were constructed by transduction using *E. coli* Keio knockouts strains. Strains, plasmids and primers are listed in Tables S3 and S4 (Supporting Information) for more details.

### Media and growth conditions

Colonies on plates grew at 37°C, in Mueller–Hinton medium (MH) media. Liquid cultures grew at 37°C in appropriate media in aerobic conditions, with 180 rotations per minute.

### Transposon insertion sequencing

Libraries were prepared as previously described (Chiang and Rubin 2002, Baharoglu et al. 2014). To achieve a library size of 600 000 clones, and subjected to passaging in MH and MH+TOB 0.5 or MH+CIP 0001 for 16 generations (Negro et al. 2019). A saturated mariner mutant library was generated by conjugation of plasmid pSC1819 from *E .coli* to *V. cholerae* WT. Briefly, pSC189 (Chiang and Rubin 2002, Baharoglu et al. 2014) was delivered from *E. coli* strain 7257 (β2163 pSC189:: spec, laboratory collection) into the *V. cholerae* WT strain. Conjugation was performed for 2 h on 0.45 µM filters. The filter was resuspended in 2 ml of MH broth. Petri dishes containing 100 µg/ml spectinomycin were then spread. The colonies were scraped and resuspended in 2 ml of MH. When sufficient single mutants were obtained (> 600 000 for 6X coverage of nonessential regions), a portion of the library was used for gDNA extraction using Qiagen DNeasy® Blood and Tissue Kit as per manufacturer’s instructions. This was used for library validation through insert amplification by nested PCR using a degenerate primer (ARB6), which contains 20 defined nucleotides followed by a randomized sequence. This was combined with a primer anchored in the edge of the transposon sequence (MV288; Baharoglu et al. 2014, Negro et al. 2019). After this, primer ARB3, which contains the first 20 nucleotides of ARB6 was used for nested amplification in combination with MV288. After validation, the libraries were passaged in MH media for 16 generations with or without 50%MIC of TOB or CIP, in triplicate. gDNA from time point 0 and both conditions after 16 generation passage in triplicate was extracted. Sequencing libraries were prepared using Agilent’s SureSelect XT2 Kit with custom RNA baits designed to hybridize the edges of the Mariner transposon. The 100 ng protocol was followed as per manufacturer’s instructions. A total of 12 cycles were used for library amplification. Agilent’s 2100 Bioanalzyer was used to verify the size of the pooled libraries and their concentration. HiSeq Paired-end Illumina sequencing technology was used producing 2 × 125 bp long reads. Reads were then filtered through transposon mapping to ensure the presence of an informative transposon/genome junction using a previously described mapping algorithm (Pierle et al. 2014). Informative reads were extracted and mapped. Reads were counted when the junction was reported as mapped inside the CDS of a gene plus an additional 50 bp upstream and downstream. Expansion or decrease of fitness of mutants was calculated in fold changes with normalized insertion numbers. Normalization calculations were applied according to van Opijnen et al. (2009). Expansion or decrease of fitness of mutants was calculated in fold changes with normalized insertion numbers. Baggerly’s test on proportions (Baggerly et al. 2003) was used to determine statistical significance as well as a Bonferroni correction for multiple hypotheses testing.

### GO enrichment analysis

GO enrichment analyses were performed on http://geneontology.org/as follows: binomial test was used to determine whether a group of gene in the tested list was more or less enriched than expected in a reference group. The annotation dataset used for the analysis was GO biological process complete.

The analyzed lists were for each antibiotic (TOB/CIP), genes (plotted on Fig. 1) with at least 2-fold change in TN-seq data at 16 generations compared to nontreated condition, and with an adjusted (Bonferroni correction) *P*-value < .05. The total number of uploaded genes list to be analyzed were 449 genes for TOB and 1004 genes for CIP.

The reference gene list was *V. cholerae* (all genes in database), 3782 genes. Annotation version: PANTHER Overrepresentation Test (Released 20220712). GO Ontology database DOI: 10.5281/zenodo.6399963 Released 2022-03-22.

### Competition experiments

Overnight cultures from single colonies of mutant lacZ+ and WT lacZ− strains were washed in phosphate buffer saline (PBS) and mixed 1:1 (500 μl + 500 μl). At this point, 100 μl of the mix were serial diluted and plated on MH agar supplemented with X-gal (5-bromo-4-chloro-3-indolyl-β-D-galactopyranoside) at 40 μg/ml to assess T0 initial 1:1 ratio. At the same time, 10 μl from the mix were added to 2 ml (approximately 5 × 10^5^ cells/ml) of MH or MH supplemented with sub-MIC antibiotics (TCL: triclosan 0.01 µM, TOB: tobramycin 0.6 μg/ml; GEN: gentamicin 0.5 μg/ml; CIP: ciprofloxacin 0.002 µg/ml, CM: chloramphenicol 0.4 µg/ml, TRM: trimethoprim 0.4 µg/ml, and CRB: carbenicillin 2.5 µg/ml) and incubated with agitation at 37°C for 20 h, which corresponds to nine generations. Cultures were then diluted and plated on MH agar plates supplemented with X-gal. Plates were incubated overnight at 37°C and the number of blue and white CFUs was assessed. Competitive index was calculated by dividing the number of blue CFUs (lacZ+ strain) by the number of white CFUs (lacZ− strain) and normalizing this ratio to the T0 initial ratio.

### MIC determination

Stationary phase cultures grown in MH were diluted 20 times in PBS, and 300 µl were plated on MH plates and dried for 10 min. Etests (Biomérieux) were placed on the plates and incubated overnight at 37°C. The Etest for ampicillin (AMP) was used for the evaluation of the MIC of CRB.

Survival/tolerance tests were performed on early exponential phase cultures. In order to clear the culture from previously nongrowing cells that could potentially be present from the stationary phase inoculum, we performed a two-step dilution protocol, before antibiotic treatment. Overnight *V. cholerae* cultures were first diluted 1000x in 4 ml fresh MH medium, and incubated at 37°C with shaking. When the OD 620 nm reached ∼0.2, cultures were diluted 1000x a second time, in order to clear them from nongrowing cells, in Erlenmeyers containing 25 ml fresh MH medium, and were allowed to grow at 37°C. When cultures reached an OD 620 nm between 0.25 and 0.3 (early exponential phase), appropriate dilutions were plated on MH plates to determine the total number of CFUs in time zero untreated cultures. Note that for *V. cholerae*, it was important to treat cultures at the precise OD 620 nm 0.25–0.3, as persistence levels seem to be particularly sensitive to growth phase in this species, where they decline in stationary phase. a volume of 5 ml of cultures were collected into 50 ml Falcon tubes and treated with lethal doses of desired antibiotics (10 times the MIC: TOB 10 µg/ml, CRB 50 µg/ml, CIP 0.025 µg/ml, and TRM 5 µg/ml) for 20 h at 37°C with shaking in order to guarantee oxygenation. Appropriate dilutions were then plated on MH agar without antibiotics and proportion of growing CFUs were calculated by doing a ratio with total CFUs at time zero. Experiments were performed three to eight times.

### RNA purification and analysis of rRNA species

For RNA extraction, overnight cultures were diluted 1:1000 in MH medium and grown with agitation at 37°C until an OD600 of 0.3 (exponential phase). 0.5 ml of these cultures were centrifuged and supernatant removed. Pellets were homogenized by resuspension with 1.5 ml of cold TRIzol Reagent. Next, 300 μl chloroform were added to the samples following mix by vortexing. Samples were then centrifuged at 4°C for 10 min. Upper (aqueous) phase was transferred to a new 2 ml tube and mixed with 1 volume of 70% ethanol. From this point, the homogenate was loaded into a RNeasy Mini kit (Qiagen) column and RNA purification proceeded according to the manufacturer’s instructions. Samples were then subjected to DNase treatment using TURBO DNA-free Kit (Ambion) according to the manufacturer’s instructions. Total RNA samples were then analyzed on an Agilent 2100 Bioanalyzer (Agilent Technologies) using the Agilent RNA 6000 nano kit according to the instructions of the manufacturer.

### Rifampicin spontaneous mutation tests

Stationary phase cultures were plated in parallel on MH and MH plate supplemented with RIF: rifampicin 1 µg/ml. The mutation frequency was calculated as CFU MH + RIF/total CFU on MH.

### Growth of *E. coli* on microtiter plate reader

Overnight cultures were diluted 100x in fresh MH medium, on 96-well plates. Each well contained 200 µl. Plates were incubated with shaking on TECAN plate reader device at 37°C, OD 620 nm was measured every 15 min. Antibiotics were used at sub-MIC for MG1655 in MH liquid culture: TOB 0.2 and 0.4 µg/ml, CRB 3 µg/ml, TRM 0.15 µg/ml, GEN 0.3 and 0.4 µg/ml, and CIP 0.006 µg/ml.

### Stringent response measurements

*P1rrnB-gfp* fusion was constructed using *gfp* ASV (Andersen et al. 1998), and cloned into plasmid pSC101. *P1rrnB-GFPasv* transcriptional fusion was amplified from strain R438 (*E. coli* MG1655 *attB:: P1rrnB gfp-ASV:: kan* provided by Ivan Matic) using primers AFC060 and AFC055, thus including 42 bp upstream of *rrnB* transcription initiation site. PCR product was then cloned in pTOPOblunt vector and subcloned to pSC101 by EcoRI digestion and ligation. The final construct was confirmed by sanger sequencing. The plasmid was then introduced by electroporation into the tested strains. Overnight cultures were performed in MH + CRB 100 µg/ml and diluted 500x in 10 ml fresh MH, in an Erlenmeyer. At time points 0 min, and every 30 during 3 h, the OD 620 nm was measured and fluorescence was quantified in flow cytometry. For each experiment, 50 000–100 000 events were counted on the Miltenyi MACSquant device.

### UV sensitivity measurements

Overnight cultures were diluted 1:100 in MH medium and grown with agitation at 37°C until an OD 600 nm of 0.5–0.7. Appropriate dilutions were then plated on MH agar. The proportion of growing CFUs after irradiation at 60 Joules over total population before irradiation was calculated, doing a ratio with total CFUs. Experiments were performed three to eight times.

### Quantification and statistical analysis

First an F-test was performed in order to determine whether variances are equal or different between comparisons. For comparisons with equal variance, Student’s *t*-test was used. For comparisons with significantly different variances, we used Welch’s *t*-test. For multiple comparisons, we used one-way ANOVA. We used GraphPad Prism to determine the statistical differences (*P*-value) between groups. **** means *P*< .0001, *** means *P*< .001, ** means *P*< .01, and * means *P*< .05. For survival tests, data were first log transformed in order to achieve normal distribution, and statistical tests were performed on these log-transformed data. Number of replicates for each experiment was 3 < n < 6. Means and geometric means for logarithmic values were also calculated using GraphPad Prism. For persistence tests, data were first log transformed in order to achieve normal distribution, and statistical tests were performed on these log-transformed data.

## Supplementary Material

uqac019_Supplemental_Files

## Data Availability

TN-seq data is available through the GEO accession number: GSE198341 for TOB and accession numbers SRR8351961, SRR8351962, SRR8351957, SRR8351958, SRR8351959, SRR8351960, SRR8351965, SRR8351966, SRR8351963, SRR8351964, SRR8351967, and SRR8351968 for CIP.
