# Supplementary material for: Nonessential tRNA and rRNA modifications impact the bacterial response to sub-MIC antibiotic stress
Source: Microlife. 2022 Sep 14;3:uqac019. doi: 10.1093/femsml/uqac019 (PMC10117853; doi:10.1093/femsml/uqac019)
Supplement: uqac019_Supplemental_Files [file uqac019_supplemental_files.zip › TableS2_supplementary_data.docx]

**Table S2. Gene ontology enrichment analysis for gene with at least 2-fold change in TN-seq data at 16 generations compared to non-treated condition, and with an adjusted (Bonferroni correction) p-value <0,05.**

| **GO biological process complete**  **TOB**  **16 gen TOB vs 16 gen MH** | **Vibrio cholerae REFLIST (3782)** | **upload_1 (447)** | **expected** | **fold overrepresentation** | **P-value** |
| --- | --- | --- | --- | --- | --- |
| regulation of proteolysis (GO:0030162) | 2 | 2 | .24 | 8,46 | 2.39E-02 |
| regulation of secondary metabolite biosynthetic process (GO:1900376) | 2 | 2 | .24 | 8,46 | 2.39E-02 |
| phenylalanyl-tRNA aminoacylation (GO:0006432) | 2 | 2 | .24 | 8,46 | 2.39E-02 |
| O antigen metabolic process (GO:0046402) | 2 | 2 | .24 | 8,46 | 2.39E-02 |
| enzyme-directed rRNA 2'-O-methylation (GO:0000453) | 2 | 2 | .24 | 8,46 | 2.39E-02 |
| rRNA 2'-O-methylation (GO:0000451) | 2 | 2 | .24 | 8,46 | 2.39E-02 |
| regulation of secondary metabolic process (GO:0043455) | 2 | 2 | .24 | 8,46 | 2.39E-02 |
| guanosine tetraphosphate biosynthetic process (GO:0015970) | 2 | 2 | .24 | 8,46 | 2.39E-02 |
| nicotinamide nucleotide biosynthetic process from aspartate (GO:0019355) | 2 | 2 | .24 | 8,46 | 2.39E-02 |
| purine ribonucleoside bisphosphate biosynthetic process (GO:0034036) | 2 | 2 | .24 | 8,46 | 2.39E-02 |
| O antigen biosynthetic process (GO:0009243) | 2 | 2 | .24 | 8,46 | 2.39E-02 |
| 'de novo' NAD biosynthetic process from aspartate (GO:0034628) | 2 | 2 | .24 | 8,46 | 2.39E-02 |
| 'de novo' NAD biosynthetic process (GO:0034627) | 2 | 2 | .24 | 8,46 | 2.39E-02 |
| protein transport by the Tat complex (GO:0043953) | 4 | 4 | .47 | 8,46 | 1.42E-03 |
| prosthetic group biosynthetic process (GO:0051191) | 2 | 2 | .24 | 8,46 | 2.39E-02 |
| biotin biosynthetic process (GO:0009102) | 7 | 6 | .83 | 7,25 | 2.15E-04 |
| biotin metabolic process (GO:0006768) | 7 | 6 | .83 | 7,25 | 2.15E-04 |
| cytochrome complex assembly (GO:0017004) | 10 | 7 | 1.18 | 5,92 | 2.22E-04 |
| glutamyl-tRNA aminoacylation (GO:0006424) | 3 | 2 | .35 | 5,64 | 4.97E-02 |
| tRNA dihydrouridine synthesis (GO:0002943) | 3 | 2 | .35 | 5,64 | 4.97E-02 |
| RNA (guanine-N7)-methylation (GO:0036265) | 3 | 2 | .35 | 5,64 | 4.97E-02 |
| ribonucleotide catabolic process (GO:0009261) | 3 | 2 | .35 | 5,64 | 4.97E-02 |
| vitamin transmembrane transport (GO:0035461) | 3 | 2 | .35 | 5,64 | 4.97E-02 |
| protein unfolding (GO:0043335) | 3 | 2 | .35 | 5,64 | 4.97E-02 |
| demethylation (GO:0070988) | 3 | 2 | .35 | 5,64 | 4.97E-02 |
| regulation of cellular catabolic process (GO:0031329) | 3 | 2 | .35 | 5,64 | 4.97E-02 |
| thiamine transport (GO:0015888) | 3 | 2 | .35 | 5,64 | 4.97E-02 |
| bacterial-type flagellum assembly (GO:0044780) | 11 | 7 | 1.30 | 5,38 | 3.91E-04 |
| vitamin transport (GO:0051180) | 8 | 5 | .95 | 5,29 | 2.85E-03 |
| GDP-mannose metabolic process (GO:0019673) | 5 | 3 | .59 | 5,08 | 2.21E-02 |
| cobalamin transport (GO:0015889) | 5 | 3 | .59 | 5,08 | 2.21E-02 |
| arginine catabolic process (GO:0006527) | 5 | 3 | .59 | 5,08 | 2.21E-02 |
| transcription antitermination (GO:0031564) | 7 | 4 | .83 | 4,83 | 1.01E-02 |
| regulation of protein-containing complex disassembly (GO:0043244) | 9 | 5 | 1.06 | 4,7 | 4.67E-03 |
| regulation of cellular component organization (GO:0051128) | 12 | 6 | 1.42 | 4,23 | 3.34E-03 |
| NAD biosynthetic process (GO:0009435) | 6 | 3 | .71 | 4,23 | 3.51E-02 |
| thiamine-containing compound biosynthetic process (GO:0042724) | 8 | 4 | .95 | 4,23 | 1.57E-02 |
| queuosine biosynthetic process (GO:0008616) | 8 | 4 | .95 | 4,23 | 1.57E-02 |
| regulation of DNA-templated transcription, termination (GO:0031554) | 8 | 4 | .95 | 4,23 | 1.57E-02 |
| queuosine metabolic process (GO:0046116) | 8 | 4 | .95 | 4,23 | 1.57E-02 |
| thiamine diphosphate metabolic process (GO:0042357) | 6 | 3 | .71 | 4,23 | 3.51E-02 |
| thiamine diphosphate biosynthetic process (GO:0009229) | 6 | 3 | .71 | 4,23 | 3.51E-02 |
| thiamine biosynthetic process (GO:0009228) | 8 | 4 | .95 | 4,23 | 1.57E-02 |
| primary alcohol biosynthetic process (GO:0034309) | 10 | 5 | 1.18 | 4,23 | 7.19E-03 |
| intracellular protein transmembrane transport (GO:0065002) | 8 | 4 | .95 | 4,23 | 1.57E-02 |
| bacterial-type flagellum organization (GO:0044781) | 17 | 8 | 2.01 | 3,98 | 1.09E-03 |
| cytokinetic process (GO:0032506) | 13 | 6 | 1.54 | 3,91 | 4.90E-03 |
| thiamine-containing compound metabolic process (GO:0042723) | 9 | 4 | 1.06 | 3,76 | 2.30E-02 |
| thiamine metabolic process (GO:0006772) | 9 | 4 | 1.06 | 3,76 | 2.30E-02 |
| nicotinamide nucleotide metabolic process (GO:0046496) | 9 | 4 | 1.06 | 3,76 | 2.30E-02 |
| pyridine nucleotide metabolic process (GO:0019362) | 9 | 4 | 1.06 | 3,76 | 2.30E-02 |
| alcohol biosynthetic process (GO:0046165) | 14 | 6 | 1.65 | 3,63 | 6.94E-03 |
| sodium ion transport (GO:0006814) | 28 | 12 | 3.31 | 3,63 | 1.62E-04 |
| cell septum assembly (GO:0090529) | 12 | 5 | 1.42 | 3,53 | 1.48E-02 |
| primary alcohol metabolic process (GO:0034308) | 12 | 5 | 1.42 | 3,53 | 1.48E-02 |
| cell projection assembly (GO:0030031) | 17 | 7 | 2.01 | 3,48 | 4.54E-03 |
| cell cycle process (GO:0022402) | 20 | 8 | 2.36 | 3,38 | 2.96E-03 |
| cytokinesis (GO:0000910) | 20 | 8 | 2.36 | 3,38 | 2.96E-03 |
| RNA catabolic process (GO:0006401) | 13 | 5 | 1.54 | 3,25 | 2.01E-02 |
| rRNA methylation (GO:0031167) | 21 | 8 | 2.48 | 3,22 | 3.95E-03 |
| protein-containing complex assembly (GO:0065003) | 30 | 11 | 3.55 | 3,1 | 1.07E-03 |
| rRNA base methylation (GO:0070475) | 11 | 4 | 1.30 | 3,08 | 4.29E-02 |
| protein peptidyl-prolyl isomerization (GO:0000413) | 11 | 4 | 1.30 | 3,08 | 4.29E-02 |
| sodium ion transmembrane transport (GO:0035725) | 11 | 4 | 1.30 | 3,08 | 4.29E-02 |
| RNA methylation (GO:0001510) | 36 | 13 | 4.25 | 3,06 | 4.48E-04 |
| protein-containing complex organization (GO:0043933) | 36 | 13 | 4.25 | 3,06 | 4.48E-04 |
| reproduction of a single-celled organism (GO:0032505) | 14 | 5 | 1.65 | 3,02 | 2.66E-02 |
| reproductive process (GO:0022414) | 14 | 5 | 1.65 | 3,02 | 2.66E-02 |
| FtsZ-dependent cytokinesis (GO:0043093) | 14 | 5 | 1.65 | 3,02 | 2.66E-02 |
| purine nucleoside bisphosphate biosynthetic process (GO:0034033) | 14 | 5 | 1.65 | 3,02 | 2.66E-02 |
| ribonucleoside bisphosphate biosynthetic process (GO:0034030) | 14 | 5 | 1.65 | 3,02 | 2.66E-02 |
| reproduction (GO:0000003) | 14 | 5 | 1.65 | 3,02 | 2.66E-02 |
| asexual reproduction (GO:0019954) | 14 | 5 | 1.65 | 3,02 | 2.66E-02 |
| nucleoside bisphosphate biosynthetic process (GO:0033866) | 14 | 5 | 1.65 | 3,02 | 2.66E-02 |
| cell projection organization (GO:0030030) | 23 | 8 | 2.72 | 2,94 | 6.70E-03 |
| non-membrane-bounded organelle assembly (GO:0140694) | 26 | 9 | 3.07 | 2,93 | 4.28E-03 |
| organelle assembly (GO:0070925) | 26 | 9 | 3.07 | 2,93 | 4.28E-03 |
| cellular component assembly (GO:0022607) | 81 | 28 | 9.57 | 2,92 | 6.81E-07 |
| rRNA modification (GO:0000154) | 29 | 10 | 3.43 | 2,92 | 2.74E-03 |
| macromolecule methylation (GO:0043414) | 47 | 16 | 5.55 | 2,88 | 1.98E-04 |
| pyridine-containing compound metabolic process (GO:0072524) | 15 | 5 | 1.77 | 2,82 | 3.42E-02 |
| cellular macromolecule localization (GO:0070727) | 25 | 8 | 2.95 | 2,71 | 1.07E-02 |
| cellular protein localization (GO:0034613) | 25 | 8 | 2.95 | 2,71 | 1.07E-02 |
| negative regulation of nucleic acid-templated transcription (GO:1903507) | 22 | 7 | 2.60 | 2,69 | 1.69E-02 |
| negative regulation of transcription, DNA-templated (GO:0045892) | 22 | 7 | 2.60 | 2,69 | 1.69E-02 |
| negative regulation of RNA biosynthetic process (GO:1902679) | 22 | 7 | 2.60 | 2,69 | 1.69E-02 |
| negative regulation of macromolecule biosynthetic process (GO:0010558) | 29 | 9 | 3.43 | 2,63 | 8.44E-03 |
| rRNA processing (GO:0006364) | 42 | 13 | 4.96 | 2,62 | 1.77E-03 |
| rRNA metabolic process (GO:0016072) | 42 | 13 | 4.96 | 2,62 | 1.77E-03 |
| cellular macromolecule catabolic process (GO:0044265) | 26 | 8 | 3.07 | 2,6 | 1.32E-02 |
| methylation (GO:0032259) | 65 | 20 | 7.68 | 2,6 | 1.28E-04 |
| monocarboxylic acid biosynthetic process (GO:0072330) | 39 | 12 | 4.61 | 2,6 | 2.75E-03 |
| cell cycle (GO:0007049) | 46 | 14 | 5.44 | 2,58 | 1.40E-03 |
| regulation of cellular amide metabolic process (GO:0034248) | 20 | 6 | 2.36 | 2,54 | 3.31E-02 |
| post-transcriptional regulation of gene expression (GO:0010608) | 20 | 6 | 2.36 | 2,54 | 3.31E-02 |
| negative regulation of biosynthetic process (GO:0009890) | 30 | 9 | 3.55 | 2,54 | 1.04E-02 |
| negative regulation of cellular biosynthetic process (GO:0031327) | 30 | 9 | 3.55 | 2,54 | 1.04E-02 |
| negative regulation of macromolecule metabolic process (GO:0010605) | 37 | 11 | 4.37 | 2,52 | 5.20E-03 |
| bacterial-type flagellum-dependent cell motility (GO:0071973) | 24 | 7 | 2.84 | 2,47 | 2.55E-02 |
| organic hydroxy compound biosynthetic process (GO:1901617) | 24 | 7 | 2.84 | 2,47 | 2.55E-02 |
| negative regulation of RNA metabolic process (GO:0051253) | 24 | 7 | 2.84 | 2,47 | 2.55E-02 |
| ribosome biogenesis (GO:0042254) | 59 | 17 | 6.97 | 2,44 | 8.25E-04 |
| RNA modification (GO:0009451) | 80 | 23 | 9.46 | 2,43 | 1.11E-04 |
| cellular component biogenesis (GO:0044085) | 161 | 46 | 19.03 | 2,42 | 4.99E-08 |
| ribonucleoprotein complex biogenesis (GO:0022613) | 60 | 17 | 7.09 | 2,4 | 9.87E-04 |
| negative regulation of metabolic process (GO:0009892) | 39 | 11 | 4.61 | 2,39 | 7.56E-03 |
| negative regulation of nitrogen compound metabolic process (GO:0051172) | 32 | 9 | 3.78 | 2,38 | 1.51E-02 |
| cilium or flagellum-dependent cell motility (GO:0001539) | 25 | 7 | 2.95 | 2,37 | 3.08E-02 |
| negative regulation of nucleobase-containing compound metabolic process (GO:0045934) | 25 | 7 | 2.95 | 2,37 | 3.08E-02 |
| archaeal or bacterial-type flagellum-dependent cell motility (GO:0097588) | 25 | 7 | 2.95 | 2,37 | 3.08E-02 |
| water-soluble vitamin biosynthetic process (GO:0042364) | 44 | 12 | 5.20 | 2,31 | 6.97E-03 |
| cellular component organization or biogenesis (GO:0071840) | 218 | 59 | 25.77 | 2,29 | 3.81E-09 |
| sulfur compound biosynthetic process (GO:0044272) | 52 | 14 | 6.15 | 2,28 | 4.17E-03 |
| regulation of protein metabolic process (GO:0051246) | 26 | 7 | 3.07 | 2,28 | 3.68E-02 |
| vitamin biosynthetic process (GO:0009110) | 45 | 12 | 5.32 | 2,26 | 8.23E-03 |
| cell division (GO:0051301) | 60 | 16 | 7.09 | 2,26 | 2.50E-03 |
| negative regulation of cellular metabolic process (GO:0031324) | 34 | 9 | 4.02 | 2,24 | 2.14E-02 |
| cellular component organization (GO:0016043) | 163 | 43 | 19.27 | 2,23 | 1.13E-06 |
| establishment of protein localization (GO:0045184) | 77 | 20 | 9.10 | 2,2 | 1.05E-03 |
| alcohol metabolic process (GO:0006066) | 27 | 7 | 3.19 | 2,19 | 4.35E-02 |
| protein localization (GO:0008104) | 78 | 20 | 9.22 | 2,17 | 1.23E-03 |
| RNA processing (GO:0006396) | 103 | 26 | 12.17 | 2,14 | 3.06E-04 |
| organelle organization (GO:0006996) | 68 | 17 | 8.04 | 2,12 | 3.56E-03 |
| ncRNA processing (GO:0034470) | 99 | 24 | 11.70 | 2,05 | 8.95E-04 |
| protein transport (GO:0015031) | 71 | 17 | 8.39 | 2,03 | 5.41E-03 |
| phospholipid biosynthetic process (GO:0008654) | 38 | 9 | 4.49 | 2 | 3.90E-02 |
| phospholipid metabolic process (GO:0006644) | 38 | 9 | 4.49 | 2 | 3.90E-02 |
| water-soluble vitamin metabolic process (GO:0006767) | 51 | 12 | 6.03 | 1,99 | 2.00E-02 |
| ncRNA metabolic process (GO:0034660) | 124 | 29 | 14.66 | 1,98 | 4.79E-04 |
| vitamin metabolic process (GO:0006766) | 52 | 12 | 6.15 | 1,95 | 2.27E-02 |
| nucleobase-containing compound catabolic process (GO:0034655) | 39 | 9 | 4.61 | 1,95 | 4.47E-02 |
| tRNA modification (GO:0006400) | 48 | 11 | 5.67 | 1,94 | 2.97E-02 |
| tRNA processing (GO:0008033) | 63 | 14 | 7.45 | 1,88 | 1.95E-02 |
| metal ion transport (GO:0030001) | 78 | 17 | 9.22 | 1,84 | 1.28E-02 |
| tRNA metabolic process (GO:0006399) | 88 | 19 | 10.40 | 1,83 | 9.68E-03 |
| RNA metabolic process (GO:0016070) | 186 | 39 | 21.98 | 1,77 | 4.66E-04 |
| macromolecule modification (GO:0043412) | 202 | 42 | 23.87 | 1,76 | 3.35E-04 |
| macromolecule localization (GO:0033036) | 102 | 21 | 12.06 | 1,74 | 1.11E-02 |
| lipid biosynthetic process (GO:0008610) | 78 | 16 | 9.22 | 1,74 | 2.53E-02 |
| regulation of gene expression (GO:0010468) | 247 | 46 | 29.19 | 1,58 | 1.72E-03 |
| regulation of macromolecule metabolic process (GO:0060255) | 264 | 49 | 31.20 | 1,57 | 1.30E-03 |
| monocarboxylic acid metabolic process (GO:0032787) | 109 | 20 | 12.88 | 1,55 | 3.73E-02 |
| regulation of metabolic process (GO:0019222) | 269 | 49 | 31.79 | 1,54 | 1.91E-03 |
| gene expression (GO:0010467) | 264 | 48 | 31.20 | 1,54 | 2.20E-03 |
| nitrogen compound transport (GO:0071705) | 176 | 32 | 20.80 | 1,54 | 1.16E-02 |
| organophosphate biosynthetic process (GO:0090407) | 142 | 25 | 16.78 | 1,49 | 3.31E-02 |
| regulation of macromolecule biosynthetic process (GO:0010556) | 239 | 42 | 28.25 | 1,49 | 7.25E-03 |
| regulation of cellular biosynthetic process (GO:0031326) | 239 | 42 | 28.25 | 1,49 | 7.25E-03 |
| regulation of biosynthetic process (GO:0009889) | 241 | 42 | 28.48 | 1,47 | 8.30E-03 |
| regulation of nitrogen compound metabolic process (GO:0051171) | 255 | 44 | 30.14 | 1,46 | 8.22E-03 |
| organic cyclic compound biosynthetic process (GO:1901362) | 267 | 46 | 31.56 | 1,46 | 7.13E-03 |
| regulation of primary metabolic process (GO:0080090) | 256 | 44 | 30.26 | 1,45 | 8.76E-03 |
| regulation of RNA biosynthetic process (GO:2001141) | 223 | 38 | 26.36 | 1,44 | 1.62E-02 |
| heterocycle biosynthetic process (GO:0018130) | 253 | 43 | 29.90 | 1,44 | 1.14E-02 |
| regulation of cellular metabolic process (GO:0031323) | 254 | 43 | 30.02 | 1,43 | 1.21E-02 |
| regulation of RNA metabolic process (GO:0051252) | 226 | 38 | 26.71 | 1,42 | 1.95E-02 |
| regulation of transcription, DNA-templated (GO:0006355) | 221 | 37 | 26.12 | 1,42 | 2.23E-02 |
| regulation of nucleic acid-templated transcription (GO:1903506) | 221 | 37 | 26.12 | 1,42 | 2.23E-02 |
| small molecule biosynthetic process (GO:0044283) | 233 | 39 | 27.54 | 1,42 | 1.93E-02 |
| regulation of nucleobase-containing compound metabolic process (GO:0019219) | 231 | 38 | 27.30 | 1,39 | 2.62E-02 |
| cellular nitrogen compound biosynthetic process (GO:0044271) | 355 | 58 | 41.96 | 1,38 | 7.76E-03 |
| heterocycle metabolic process (GO:0046483) | 566 | 92 | 66.90 | 1,38 | 8.61E-04 |
| organic cyclic compound metabolic process (GO:1901360) | 586 | 95 | 69.26 | 1,37 | 7.57E-04 |
| aromatic compound biosynthetic process (GO:0019438) | 235 | 38 | 27.77 | 1,37 | 3.28E-02 |
| nucleic acid metabolic process (GO:0090304) | 316 | 50 | 37.35 | 1,34 | 2.23E-02 |
| cellular aromatic compound metabolic process (GO:0006725) | 557 | 87 | 65.83 | 1,32 | 3.81E-03 |
| nucleobase-containing compound metabolic process (GO:0006139) | 470 | 73 | 55.55 | 1,31 | 9.30E-03 |
| cellular nitrogen compound metabolic process (GO:0034641) | 662 | 102 | 78.24 | 1,3 | 2.49E-03 |
| organic substance biosynthetic process (GO:1901576) | 610 | 93 | 72.10 | 1,29 | 5.41E-03 |
| cellular macromolecule metabolic process (GO:0044260) | 486 | 74 | 57.44 | 1,29 | 1.37E-02 |
| regulation of biological process (GO:0050789) | 469 | 71 | 55.43 | 1,28 | 1.77E-02 |
| regulation of cellular process (GO:0050794) | 418 | 63 | 49.40 | 1,28 | 2.71E-02 |
| biosynthetic process (GO:0009058) | 624 | 94 | 73.75 | 1,27 | 7.15E-03 |
| cellular biosynthetic process (GO:0044249) | 599 | 89 | 70.80 | 1,26 | 1.26E-02 |
| organonitrogen compound biosynthetic process (GO:1901566) | 418 | 62 | 49.40 | 1,25 | 3.72E-02 |
| biological regulation (GO:0065007) | 508 | 75 | 60.04 | 1,25 | 2.50E-02 |
| cellular metabolic process (GO:0044237) | 1232 | 179 | 145.61 | 1,23 | 5.40E-04 |
| macromolecule metabolic process (GO:0043170) | 689 | 99 | 81.43 | 1,22 | 2.01E-02 |
| metabolic process (GO:0008152) | 1391 | 199 | 164.40 | 1,21 | 4.70E-04 |
| nitrogen compound metabolic process (GO:0006807) | 1032 | 146 | 121.97 | 1,2 | 6.94E-03 |
| organic substance metabolic process (GO:0071704) | 1249 | 174 | 147.62 | 1,18 | 5.03E-03 |
| cellular process (GO:0009987) | 1874 | 261 | 221.49 | 1,18 | 1.09E-04 |
| primary metabolic process (GO:0044238) | 1106 | 149 | 130.72 | 1,14 | 3.34E-02 |
| transmembrane transport (GO:0055085) | 371 | 31 | 43.85 | 0,71 | 2.09E-02 |
| carbohydrate transport (GO:0008643) | 43 | 1 | 5.08 | 0,2 | 3.70E-02 |
| **GO biological process complete**  **CIP**  **16 gen CIP vs 16 gen MH** | **Vibrio cholerae - REFLIST (3782)** | **upload_1 (1004)** | **(expected)** | **(fold overrepresentation)** | **P-value** |
| protein maturation by iron-sulfur cluster transfer (GO:0097428) | 4 | 4 | 1.06 | 3,77 | 2.29E-02 |
| tRNA threonylcarbamoyladenosine metabolic process (GO:0070525) | 3 | 3 | .80 | 3,77 | 4.68E-02 |
| organelle disassembly (GO:1903008) | 3 | 3 | .80 | 3,77 | 4.68E-02 |
| AMP biosynthetic process (GO:0006167) | 4 | 4 | 1.06 | 3,77 | 2.29E-02 |
| 'de novo' UMP biosynthetic process (GO:0044205) | 7 | 7 | 1.86 | 3,77 | 3.02E-03 |
| tRNA threonylcarbamoyladenosine modification (GO:0002949) | 3 | 3 | .80 | 3,77 | 4.68E-02 |
| DNA-templated transcription, termination (GO:0006353) | 5 | 5 | 1.33 | 3,77 | 1.15E-02 |
| lipoprotein transport (GO:0042953) | 4 | 4 | 1.06 | 3,77 | 2.29E-02 |
| glycosylation (GO:0070085) | 3 | 3 | .80 | 3,77 | 4.68E-02 |
| AMP metabolic process (GO:0046033) | 4 | 4 | 1.06 | 3,77 | 2.29E-02 |
| pantothenate biosynthetic process (GO:0015940) | 3 | 3 | .80 | 3,77 | 4.68E-02 |
| pantothenate metabolic process (GO:0015939) | 4 | 4 | 1.06 | 3,77 | 2.29E-02 |
| ribosomal small subunit assembly (GO:0000028) | 5 | 5 | 1.33 | 3,77 | 1.15E-02 |
| nucleoside monophosphate phosphorylation (GO:0046940) | 4 | 4 | 1.06 | 3,77 | 2.29E-02 |
| regulation of DNA replication (GO:0006275) | 3 | 3 | .80 | 3,77 | 4.68E-02 |
| protein transport by the Tat complex (GO:0043953) | 4 | 4 | 1.06 | 3,77 | 2.29E-02 |
| ribosome disassembly (GO:0032790) | 3 | 3 | .80 | 3,77 | 4.68E-02 |
| cytoplasmic translation (GO:0002181) | 4 | 4 | 1.06 | 3,77 | 2.29E-02 |
| lipid-linked peptidoglycan transport (GO:0015836) | 3 | 3 | .80 | 3,77 | 4.68E-02 |
| tRNA wobble position uridine thiolation (GO:0002143) | 5 | 5 | 1.33 | 3,77 | 1.15E-02 |
| aminoacyl-tRNA metabolism involved in translational fidelity (GO:0106074) | 7 | 6 | 1.86 | 3,23 | 1.19E-02 |
| tRNA thio-modification (GO:0034227) | 7 | 6 | 1.86 | 3,23 | 1.19E-02 |
| ribosome assembly (GO:0042255) | 14 | 12 | 3.72 | 3,23 | 4.74E-04 |
| pyridoxine biosynthetic process (GO:0008615) | 6 | 5 | 1.59 | 3,14 | 2.32E-02 |
| pyridoxine metabolic process (GO:0008614) | 6 | 5 | 1.59 | 3,14 | 2.32E-02 |
| regulation of DNA metabolic process (GO:0051052) | 6 | 5 | 1.59 | 3,14 | 2.32E-02 |
| ribonucleoprotein complex subunit organization (GO:0071826) | 12 | 10 | 3.19 | 3,14 | 1.67E-03 |
| cell septum assembly (GO:0090529) | 12 | 10 | 3.19 | 3,14 | 1.67E-03 |
| lipooligosaccharide biosynthetic process (GO:1901271) | 12 | 10 | 3.19 | 3,14 | 1.67E-03 |
| lipooligosaccharide metabolic process (GO:1901269) | 12 | 10 | 3.19 | 3,14 | 1.67E-03 |
| vitamin B6 biosynthetic process (GO:0042819) | 6 | 5 | 1.59 | 3,14 | 2.32E-02 |
| vitamin B6 metabolic process (GO:0042816) | 6 | 5 | 1.59 | 3,14 | 2.32E-02 |
| ribonucleoprotein complex assembly (GO:0022618) | 12 | 10 | 3.19 | 3,14 | 1.67E-03 |
| lipid A metabolic process (GO:0046493) | 11 | 9 | 2.92 | 3,08 | 3.15E-03 |
| lipid A biosynthetic process (GO:0009245) | 11 | 9 | 2.92 | 3,08 | 3.15E-03 |
| cytochrome complex assembly (GO:0017004) | 10 | 8 | 2.65 | 3,01 | 5.94E-03 |
| regulation of translational fidelity (GO:0006450) | 10 | 8 | 2.65 | 3,01 | 5.94E-03 |
| translational initiation (GO:0006413) | 5 | 4 | 1.33 | 3,01 | 4.58E-02 |
| Gram-negative-bacterium-type cell outer membrane assembly (GO:0043165) | 10 | 8 | 2.65 | 3,01 | 5.94E-03 |
| cell envelope organization (GO:0043163) | 10 | 8 | 2.65 | 3,01 | 5.94E-03 |
| membrane assembly (GO:0071709) | 10 | 8 | 2.65 | 3,01 | 5.94E-03 |
| division septum assembly (GO:0000917) | 10 | 8 | 2.65 | 3,01 | 5.94E-03 |
| membrane biogenesis (GO:0044091) | 10 | 8 | 2.65 | 3,01 | 5.94E-03 |
| membrane lipid biosynthetic process (GO:0046467) | 14 | 11 | 3.72 | 2,96 | 1.59E-03 |
| reproduction of a single-celled organism (GO:0032505) | 14 | 11 | 3.72 | 2,96 | 1.59E-03 |
| reproductive process (GO:0022414) | 14 | 11 | 3.72 | 2,96 | 1.59E-03 |
| FtsZ-dependent cytokinesis (GO:0043093) | 14 | 11 | 3.72 | 2,96 | 1.59E-03 |
| reproduction (GO:0000003) | 14 | 11 | 3.72 | 2,96 | 1.59E-03 |
| glycolipid biosynthetic process (GO:0009247) | 14 | 11 | 3.72 | 2,96 | 1.59E-03 |
| asexual reproduction (GO:0019954) | 14 | 11 | 3.72 | 2,96 | 1.59E-03 |
| ATP biosynthetic process (GO:0006754) | 9 | 7 | 2.39 | 2,93 | 1.12E-02 |
| proton motive force-driven ATP synthesis (GO:0015986) | 9 | 7 | 2.39 | 2,93 | 1.12E-02 |
| ribosomal small subunit biogenesis (GO:0042274) | 9 | 7 | 2.39 | 2,93 | 1.12E-02 |
| chromosome segregation (GO:0007059) | 9 | 7 | 2.39 | 2,93 | 1.12E-02 |
| cytokinetic process (GO:0032506) | 13 | 10 | 3.45 | 2,9 | 2.95E-03 |
| regulation of cell shape (GO:0008360) | 30 | 23 | 7.96 | 2,89 | 9.51E-06 |
| regulation of cell morphogenesis (GO:0022604) | 30 | 23 | 7.96 | 2,89 | 9.51E-06 |
| regulation of anatomical structure morphogenesis (GO:0022603) | 30 | 23 | 7.96 | 2,89 | 9.51E-06 |
| translation (GO:0006412) | 109 | 82 | 28.94 | 2,83 | 1.40E-16 |
| nucleoside triphosphate biosynthetic process (GO:0009142) | 16 | 12 | 4.25 | 2,83 | 1.47E-03 |
| iron-sulfur cluster assembly (GO:0016226) | 12 | 9 | 3.19 | 2,83 | 5.47E-03 |
| metallo-sulfur cluster assembly (GO:0031163) | 12 | 9 | 3.19 | 2,83 | 5.47E-03 |
| cell cycle process (GO:0022402) | 20 | 15 | 5.31 | 2,83 | 4.01E-04 |
| 'de novo' pyrimidine nucleobase biosynthetic process (GO:0006207) | 8 | 6 | 2.12 | 2,83 | 2.13E-02 |
| cytokinesis (GO:0000910) | 20 | 15 | 5.31 | 2,83 | 4.01E-04 |
| regulation of developmental process (GO:0050793) | 31 | 23 | 8.23 | 2,79 | 1.58E-05 |
| glycolipid metabolic process (GO:0006664) | 15 | 11 | 3.98 | 2,76 | 2.68E-03 |
| purine nucleoside triphosphate biosynthetic process (GO:0009145) | 11 | 8 | 2.92 | 2,74 | 1.02E-02 |
| pyrimidine nucleoside monophosphate biosynthetic process (GO:0009130) | 11 | 8 | 2.92 | 2,74 | 1.02E-02 |
| isoprenoid metabolic process (GO:0006720) | 11 | 8 | 2.92 | 2,74 | 1.02E-02 |
| isoprenoid biosynthetic process (GO:0008299) | 11 | 8 | 2.92 | 2,74 | 1.02E-02 |
| purine ribonucleoside triphosphate biosynthetic process (GO:0009206) | 11 | 8 | 2.92 | 2,74 | 1.02E-02 |
| purine ribonucleoside triphosphate metabolic process (GO:0009205) | 11 | 8 | 2.92 | 2,74 | 1.02E-02 |
| tRNA aminoacylation for protein translation (GO:0006418) | 25 | 18 | 6.64 | 2,71 | 1.82E-04 |
| tRNA aminoacylation (GO:0043039) | 25 | 18 | 6.64 | 2,71 | 1.82E-04 |
| cell cycle (GO:0007049) | 46 | 33 | 12.21 | 2,7 | 5.22E-07 |
| transcription antitermination (GO:0031564) | 7 | 5 | 1.86 | 2,69 | 4.06E-02 |
| isopentenyl diphosphate metabolic process (GO:0046490) | 7 | 5 | 1.86 | 2,69 | 4.06E-02 |
| terpenoid metabolic process (GO:0006721) | 7 | 5 | 1.86 | 2,69 | 4.06E-02 |
| terpenoid biosynthetic process (GO:0016114) | 7 | 5 | 1.86 | 2,69 | 4.06E-02 |
| lipopolysaccharide transport (GO:0015920) | 7 | 5 | 1.86 | 2,69 | 4.06E-02 |
| membrane organization (GO:0061024) | 14 | 10 | 3.72 | 2,69 | 4.91E-03 |
| response to antibiotic (GO:0046677) | 7 | 5 | 1.86 | 2,69 | 4.06E-02 |
| ribosomal large subunit biogenesis (GO:0042273) | 7 | 5 | 1.86 | 2,69 | 4.06E-02 |
| isopentenyl diphosphate biosynthetic process (GO:0009240) | 7 | 5 | 1.86 | 2,69 | 4.06E-02 |
| ribonucleoside triphosphate biosynthetic process (GO:0009201) | 14 | 10 | 3.72 | 2,69 | 4.91E-03 |
| DNA topological change (GO:0006265) | 7 | 5 | 1.86 | 2,69 | 4.06E-02 |
| heme transport (GO:0015886) | 7 | 5 | 1.86 | 2,69 | 4.06E-02 |
| isopentenyl diphosphate biosynthetic process, methylerythritol 4-phosphate pathway (GO:0019288) | 7 | 5 | 1.86 | 2,69 | 4.06E-02 |
| peptide biosynthetic process (GO:0043043) | 116 | 82 | 30.79 | 2,66 | 4.10E-15 |
| membrane lipid metabolic process (GO:0006643) | 17 | 12 | 4.51 | 2,66 | 2.40E-03 |
| pyrimidine ribonucleoside monophosphate biosynthetic process (GO:0009174) | 10 | 7 | 2.65 | 2,64 | 1.88E-02 |
| pyrimidine nucleobase biosynthetic process (GO:0019856) | 10 | 7 | 2.65 | 2,64 | 1.88E-02 |
| tRNA wobble uridine modification (GO:0002098) | 10 | 7 | 2.65 | 2,64 | 1.88E-02 |
| UMP biosynthetic process (GO:0006222) | 10 | 7 | 2.65 | 2,64 | 1.88E-02 |
| protein-containing complex assembly (GO:0065003) | 30 | 21 | 7.96 | 2,64 | 8.14E-05 |
| amino acid activation (GO:0043038) | 26 | 18 | 6.90 | 2,61 | 2.90E-04 |
| pyrimidine ribonucleotide biosynthetic process (GO:0009220) | 13 | 9 | 3.45 | 2,61 | 8.95E-03 |
| pyrimidine nucleotide biosynthetic process (GO:0006221) | 16 | 11 | 4.25 | 2,59 | 4.32E-03 |
| cellular macromolecule biosynthetic process (GO:0034645) | 173 | 118 | 45.93 | 2,57 | 3.28E-20 |
| oligosaccharide biosynthetic process (GO:0009312) | 22 | 15 | 5.84 | 2,57 | 1.04E-03 |
| pyrimidine nucleoside monophosphate metabolic process (GO:0009129) | 12 | 8 | 3.19 | 2,51 | 1.63E-02 |
| protein maturation (GO:0051604) | 18 | 12 | 4.78 | 2,51 | 3.76E-03 |
| cellular component disassembly (GO:0022411) | 9 | 6 | 2.39 | 2,51 | 3.48E-02 |
| external encapsulating structure organization (GO:0045229) | 42 | 28 | 11.15 | 2,51 | 1.39E-05 |
| protein-containing complex organization (GO:0043933) | 36 | 24 | 9.56 | 2,51 | 5.54E-05 |
| ribonucleoside triphosphate metabolic process (GO:0009199) | 15 | 10 | 3.98 | 2,51 | 7.77E-03 |
| amide biosynthetic process (GO:0043604) | 143 | 94 | 37.96 | 2,48 | 2.76E-15 |
| peptide metabolic process (GO:0006518) | 132 | 86 | 35.04 | 2,45 | 7.34E-14 |
| lipopolysaccharide biosynthetic process (GO:0009103) | 20 | 13 | 5.31 | 2,45 | 3.25E-03 |
| cell division (GO:0051301) | 60 | 39 | 15.93 | 2,45 | 5.72E-07 |
| ribonucleotide biosynthetic process (GO:0009260) | 58 | 37 | 15.40 | 2,4 | 1.69E-06 |
| pyrimidine ribonucleoside monophosphate metabolic process (GO:0009173) | 11 | 7 | 2.92 | 2,4 | 2.94E-02 |
| lipopolysaccharide metabolic process (GO:0008653) | 22 | 14 | 5.84 | 2,4 | 2.78E-03 |
| lipopolysaccharide core region metabolic process (GO:0046401) | 11 | 7 | 2.92 | 2,4 | 2.94E-02 |
| liposaccharide metabolic process (GO:1903509) | 33 | 21 | 8.76 | 2,4 | 2.90E-04 |
| UMP metabolic process (GO:0046049) | 11 | 7 | 2.92 | 2,4 | 2.94E-02 |
| lipopolysaccharide core region biosynthetic process (GO:0009244) | 11 | 7 | 2.92 | 2,4 | 2.94E-02 |
| pyrimidine nucleobase metabolic process (GO:0006206) | 11 | 7 | 2.92 | 2,4 | 2.94E-02 |
| cellular component assembly (GO:0022607) | 81 | 51 | 21.50 | 2,37 | 2.91E-08 |
| nucleoside monophosphate metabolic process (GO:0009123) | 40 | 25 | 10.62 | 2,35 | 1.06E-04 |
| nucleobase biosynthetic process (GO:0046112) | 16 | 10 | 4.25 | 2,35 | 1.18E-02 |
| cell wall organization (GO:0071555) | 32 | 20 | 8.49 | 2,35 | 4.98E-04 |
| macromolecule biosynthetic process (GO:0009059) | 238 | 148 | 63.18 | 2,34 | 1.49E-21 |
| cellular component macromolecule biosynthetic process (GO:0070589) | 34 | 21 | 9.03 | 2,33 | 4.25E-04 |
| glycosaminoglycan biosynthetic process (GO:0006024) | 34 | 21 | 9.03 | 2,33 | 4.25E-04 |
| aminoglycan biosynthetic process (GO:0006023) | 34 | 21 | 9.03 | 2,33 | 4.25E-04 |
| peptidoglycan biosynthetic process (GO:0009252) | 34 | 21 | 9.03 | 2,33 | 4.25E-04 |
| cell wall macromolecule biosynthetic process (GO:0044038) | 34 | 21 | 9.03 | 2,33 | 4.25E-04 |
| ribose phosphate biosynthetic process (GO:0046390) | 60 | 37 | 15.93 | 2,32 | 3.61E-06 |
| ribonucleoside monophosphate biosynthetic process (GO:0009156) | 33 | 20 | 8.76 | 2,28 | 7.23E-04 |
| purine nucleotide biosynthetic process (GO:0006164) | 48 | 29 | 12.74 | 2,28 | 5.60E-05 |
| ribonucleoside monophosphate metabolic process (GO:0009161) | 35 | 21 | 9.29 | 2,26 | 6.12E-04 |
| purine ribonucleotide biosynthetic process (GO:0009152) | 45 | 27 | 11.95 | 2,26 | 1.11E-04 |
| nucleoside triphosphate metabolic process (GO:0009141) | 20 | 12 | 5.31 | 2,26 | 8.34E-03 |
| nucleoside monophosphate biosynthetic process (GO:0009124) | 35 | 21 | 9.29 | 2,26 | 6.12E-04 |
| pyrimidine ribonucleotide metabolic process (GO:0009218) | 15 | 9 | 3.98 | 2,26 | 2.06E-02 |
| pyrimidine nucleotide metabolic process (GO:0006220) | 20 | 12 | 5.31 | 2,26 | 8.34E-03 |
| nucleotide biosynthetic process (GO:0009165) | 77 | 46 | 20.44 | 2,25 | 5.81E-07 |
| oligosaccharide metabolic process (GO:0009311) | 27 | 16 | 7.17 | 2,23 | 2.91E-03 |
| purine ribonucleoside monophosphate metabolic process (GO:0009167) | 22 | 13 | 5.84 | 2,23 | 7.00E-03 |
| purine nucleoside monophosphate metabolic process (GO:0009126) | 22 | 13 | 5.84 | 2,23 | 7.00E-03 |
| carbohydrate derivative biosynthetic process (GO:1901137) | 149 | 88 | 39.55 | 2,22 | 6.68E-12 |
| nucleobase metabolic process (GO:0009112) | 17 | 10 | 4.51 | 2,22 | 1.72E-02 |
| protein localization to membrane (GO:0072657) | 17 | 10 | 4.51 | 2,22 | 1.72E-02 |
| localization within membrane (GO:0051668) | 17 | 10 | 4.51 | 2,22 | 1.72E-02 |
| regulation of cellular component organization (GO:0051128) | 12 | 7 | 3.19 | 2,2 | 4.35E-02 |
| peptidoglycan-based cell wall biogenesis (GO:0009273) | 36 | 21 | 9.56 | 2,2 | 8.67E-04 |
| cell wall biogenesis (GO:0042546) | 36 | 21 | 9.56 | 2,2 | 8.67E-04 |
| cell wall macromolecule metabolic process (GO:0044036) | 36 | 21 | 9.56 | 2,2 | 8.67E-04 |
| nucleoside phosphate biosynthetic process (GO:1901293) | 79 | 46 | 20.97 | 2,19 | 1.15E-06 |
| purine-containing compound biosynthetic process (GO:0072522) | 50 | 29 | 13.27 | 2,18 | 1.12E-04 |
| nucleoside diphosphate metabolic process (GO:0009132) | 19 | 11 | 5.04 | 2,18 | 1.43E-02 |
| phospholipid biosynthetic process (GO:0008654) | 38 | 22 | 10.09 | 2,18 | 7.29E-04 |
| regulation of translation (GO:0006417) | 19 | 11 | 5.04 | 2,18 | 1.43E-02 |
| phospholipid metabolic process (GO:0006644) | 38 | 22 | 10.09 | 2,18 | 7.29E-04 |
| purine ribonucleoside monophosphate biosynthetic process (GO:0009168) | 21 | 12 | 5.57 | 2,15 | 1.19E-02 |
| purine nucleoside monophosphate biosynthetic process (GO:0009127) | 21 | 12 | 5.57 | 2,15 | 1.19E-02 |
| SOS response (GO:0009432) | 14 | 8 | 3.72 | 2,15 | 3.57E-02 |
| tRNA wobble base modification (GO:0002097) | 14 | 8 | 3.72 | 2,15 | 3.57E-02 |
| purine nucleoside bisphosphate biosynthetic process (GO:0034033) | 14 | 8 | 3.72 | 2,15 | 3.57E-02 |
| ribonucleoside bisphosphate biosynthetic process (GO:0034030) | 14 | 8 | 3.72 | 2,15 | 3.57E-02 |
| nucleoside bisphosphate biosynthetic process (GO:0033866) | 14 | 8 | 3.72 | 2,15 | 3.57E-02 |
| cellular component organization (GO:0016043) | 163 | 93 | 43.27 | 2,15 | 1.02E-11 |
| DNA conformation change (GO:0071103) | 23 | 13 | 6.11 | 2,13 | 9.86E-03 |
| cellular amide metabolic process (GO:0043603) | 181 | 102 | 48.05 | 2,12 | 1.91E-12 |
| ribonucleoside diphosphate metabolic process (GO:0009185) | 16 | 9 | 4.25 | 2,12 | 2.94E-02 |
| pyrimidine-containing compound biosynthetic process (GO:0072528) | 25 | 14 | 6.64 | 2,11 | 8.19E-03 |
| organophosphate biosynthetic process (GO:0090407) | 142 | 79 | 37.70 | 2,1 | 1.23E-09 |
| lipid biosynthetic process (GO:0008610) | 78 | 43 | 20.71 | 2,08 | 9.52E-06 |
| regulation of cellular amide metabolic process (GO:0034248) | 20 | 11 | 5.31 | 2,07 | 1.99E-02 |
| post-transcriptional regulation of gene expression (GO:0010608) | 20 | 11 | 5.31 | 2,07 | 1.99E-02 |
| fatty acid biosynthetic process (GO:0006633) | 20 | 11 | 5.31 | 2,07 | 1.99E-02 |
| ATP metabolic process (GO:0046034) | 33 | 18 | 8.76 | 2,05 | 3.91E-03 |
| DNA-templated DNA replication (GO:0006261) | 22 | 12 | 5.84 | 2,05 | 1.64E-02 |
| gene expression (GO:0010467) | 264 | 143 | 70.08 | 2,04 | 8.32E-16 |
| ribonucleotide metabolic process (GO:0009259) | 85 | 46 | 22.56 | 2,04 | 7.47E-06 |
| cellular component biogenesis (GO:0044085) | 161 | 87 | 42.74 | 2,04 | 6.82E-10 |
| cellular nitrogen compound biosynthetic process (GO:0044271) | 355 | 190 | 94.24 | 2,02 | 1.65E-20 |
| purine ribonucleoside diphosphate metabolic process (GO:0009179) | 15 | 8 | 3.98 | 2,01 | 4.97E-02 |
| purine nucleoside triphosphate metabolic process (GO:0009144) | 15 | 8 | 3.98 | 2,01 | 4.97E-02 |
| purine nucleoside diphosphate metabolic process (GO:0009135) | 15 | 8 | 3.98 | 2,01 | 4.97E-02 |
| RNA phosphodiester bond hydrolysis (GO:0090501) | 19 | 10 | 5.04 | 1,98 | 3.31E-02 |
| DNA replication (GO:0006260) | 44 | 23 | 11.68 | 1,97 | 2.07E-03 |
| cellular macromolecule localization (GO:0070727) | 25 | 13 | 6.64 | 1,96 | 1.83E-02 |
| cellular protein localization (GO:0034613) | 25 | 13 | 6.64 | 1,96 | 1.83E-02 |
| chromosome organization (GO:0051276) | 31 | 16 | 8.23 | 1,94 | 1.02E-02 |
| purine ribonucleotide metabolic process (GO:0009150) | 70 | 36 | 18.58 | 1,94 | 1.88E-04 |
| cell wall organization or biogenesis (GO:0071554) | 43 | 22 | 11.42 | 1,93 | 3.28E-03 |
| ribose phosphate metabolic process (GO:0019693) | 90 | 46 | 23.89 | 1,93 | 2.98E-05 |
| cellular component organization or biogenesis (GO:0071840) | 218 | 111 | 57.87 | 1,92 | 8.06E-11 |
| purine nucleotide metabolic process (GO:0006163) | 77 | 39 | 20.44 | 1,91 | 1.40E-04 |
| nucleobase-containing compound biosynthetic process (GO:0034654) | 162 | 82 | 43.01 | 1,91 | 3.62E-08 |
| non-membrane-bounded organelle assembly (GO:0140694) | 26 | 13 | 6.90 | 1,88 | 2.41E-02 |
| organelle assembly (GO:0070925) | 26 | 13 | 6.90 | 1,88 | 2.41E-02 |
| regulation of cellular protein metabolic process (GO:0032268) | 24 | 12 | 6.37 | 1,88 | 2.94E-02 |
| cellular lipid metabolic process (GO:0044255) | 101 | 50 | 26.81 | 1,86 | 3.05E-05 |
| regulation of biological quality (GO:0065008) | 84 | 41 | 22.30 | 1,84 | 2.05E-04 |
| cellular protein metabolic process (GO:0044267) | 239 | 116 | 63.45 | 1,83 | 4.77E-10 |
| ribonucleoprotein complex biogenesis (GO:0022613) | 60 | 29 | 15.93 | 1,82 | 1.88E-03 |
| purine-containing compound metabolic process (GO:0072521) | 81 | 39 | 21.50 | 1,81 | 3.75E-04 |
| cellular polysaccharide biosynthetic process (GO:0033692) | 27 | 13 | 7.17 | 1,81 | 3.12E-02 |
| organonitrogen compound biosynthetic process (GO:1901566) | 418 | 201 | 110.97 | 1,81 | 9.82E-17 |
| pyruvate metabolic process (GO:0006090) | 25 | 12 | 6.64 | 1,81 | 3.81E-02 |
| carbohydrate biosynthetic process (GO:0016051) | 48 | 23 | 12.74 | 1,8 | 5.79E-03 |
| tRNA metabolic process (GO:0006399) | 88 | 42 | 23.36 | 1,8 | 2.72E-04 |
| DNA recombination (GO:0006310) | 44 | 21 | 11.68 | 1,8 | 8.45E-03 |
| ribosome biogenesis (GO:0042254) | 59 | 28 | 15.66 | 1,79 | 2.87E-03 |
| protein transmembrane transport (GO:0071806) | 34 | 16 | 9.03 | 1,77 | 2.20E-02 |
| organelle organization (GO:0006996) | 68 | 32 | 18.05 | 1,77 | 1.72E-03 |
| glycosaminoglycan metabolic process (GO:0030203) | 51 | 24 | 13.54 | 1,77 | 6.05E-03 |
| peptidoglycan metabolic process (GO:0000270) | 49 | 23 | 13.01 | 1,77 | 7.30E-03 |
| organic substance biosynthetic process (GO:1901576) | 610 | 286 | 161.94 | 1,77 | 6.03E-23 |
| carbohydrate derivative metabolic process (GO:1901135) | 242 | 113 | 64.24 | 1,76 | 6.75E-09 |
| polysaccharide biosynthetic process (GO:0000271) | 30 | 14 | 7.96 | 1,76 | 3.25E-02 |
| regulation of cellular macromolecule biosynthetic process (GO:2000112) | 30 | 14 | 7.96 | 1,76 | 3.25E-02 |
| cellular biosynthetic process (GO:0044249) | 599 | 279 | 159.02 | 1,75 | 6.84E-22 |
| cellular carbohydrate biosynthetic process (GO:0034637) | 28 | 13 | 7.43 | 1,75 | 3.97E-02 |
| aromatic compound biosynthetic process (GO:0019438) | 235 | 109 | 62.38 | 1,75 | 1.81E-08 |
| regulation of protein metabolic process (GO:0051246) | 26 | 12 | 6.90 | 1,74 | 4.85E-02 |
| biosynthetic process (GO:0009058) | 624 | 286 | 165.65 | 1,73 | 1.70E-21 |
| cellular polysaccharide metabolic process (GO:0044264) | 33 | 15 | 8.76 | 1,71 | 3.36E-02 |
| aminoglycan metabolic process (GO:0006022) | 53 | 24 | 14.07 | 1,71 | 9.38E-03 |
| organophosphate metabolic process (GO:0019637) | 208 | 94 | 55.22 | 1,7 | 5.72E-07 |
| nucleotide metabolic process (GO:0009117) | 125 | 56 | 33.18 | 1,69 | 1.43E-04 |
| heterocycle biosynthetic process (GO:0018130) | 253 | 113 | 67.16 | 1,68 | 6.76E-08 |
| cellular macromolecule metabolic process (GO:0044260) | 486 | 216 | 129.02 | 1,67 | 2.03E-14 |
| organic cyclic compound biosynthetic process (GO:1901362) | 267 | 118 | 70.88 | 1,66 | 5.83E-08 |
| establishment of protein localization (GO:0045184) | 77 | 34 | 20.44 | 1,66 | 3.39E-03 |
| pyrimidine-containing compound metabolic process (GO:0072527) | 34 | 15 | 9.03 | 1,66 | 4.16E-02 |
| monocarboxylic acid biosynthetic process (GO:0072330) | 39 | 17 | 10.35 | 1,64 | 3.48E-02 |
| protein localization (GO:0008104) | 78 | 34 | 20.71 | 1,64 | 4.11E-03 |
| nucleoside phosphate metabolic process (GO:0006753) | 129 | 56 | 34.25 | 1,64 | 3.09E-04 |
| lipid metabolic process (GO:0006629) | 120 | 52 | 31.86 | 1,63 | 5.20E-04 |
| RNA biosynthetic process (GO:0032774) | 44 | 19 | 11.68 | 1,63 | 2.91E-02 |
| ncRNA metabolic process (GO:0034660) | 124 | 53 | 32.92 | 1,61 | 6.27E-04 |
| nucleic acid-templated transcription (GO:0097659) | 40 | 17 | 10.62 | 1,6 | 4.23E-02 |
| transcription, DNA-templated (GO:0006351) | 40 | 17 | 10.62 | 1,6 | 4.23E-02 |
| cellular nitrogen compound metabolic process (GO:0034641) | 662 | 281 | 175.74 | 1,6 | 1.53E-16 |
| protein metabolic process (GO:0019538) | 330 | 140 | 87.60 | 1,6 | 3.34E-08 |
| nucleic acid phosphodiester bond hydrolysis (GO:0090305) | 52 | 22 | 13.80 | 1,59 | 2.44E-02 |
| protein transport (GO:0015031) | 71 | 30 | 18.85 | 1,59 | 1.01E-02 |
| tRNA modification (GO:0006400) | 48 | 20 | 12.74 | 1,57 | 3.51E-02 |
| RNA metabolic process (GO:0016070) | 186 | 77 | 49.38 | 1,56 | 1.09E-04 |
| electron transport chain (GO:0022900) | 58 | 24 | 15.40 | 1,56 | 2.44E-02 |
| nucleobase-containing small molecule metabolic process (GO:0055086) | 160 | 66 | 42.47 | 1,55 | 3.68E-04 |
| water-soluble vitamin metabolic process (GO:0006767) | 51 | 21 | 13.54 | 1,55 | 3.50E-02 |
| macromolecule localization (GO:0033036) | 102 | 42 | 27.08 | 1,55 | 4.17E-03 |
| nucleobase-containing compound metabolic process (GO:0006139) | 470 | 191 | 124.77 | 1,53 | 1.70E-09 |
| vitamin metabolic process (GO:0006766) | 52 | 21 | 13.80 | 1,52 | 4.14E-02 |
| nucleic acid metabolic process (GO:0090304) | 316 | 127 | 83.89 | 1,51 | 2.50E-06 |
| macromolecule metabolic process (GO:0043170) | 689 | 275 | 182.91 | 1,5 | 6.20E-13 |
| cellular aromatic compound metabolic process (GO:0006725) | 557 | 220 | 147.87 | 1,49 | 7.87E-10 |
| monocarboxylic acid metabolic process (GO:0032787) | 109 | 43 | 28.94 | 1,49 | 7.70E-03 |
| heterocycle metabolic process (GO:0046483) | 566 | 222 | 150.25 | 1,48 | 1.17E-09 |
| DNA metabolic process (GO:0006259) | 136 | 53 | 36.10 | 1,47 | 4.24E-03 |
| organic cyclic compound metabolic process (GO:1901360) | 586 | 228 | 155.56 | 1,47 | 1.30E-09 |
| generation of precursor metabolites and energy (GO:0006091) | 107 | 41 | 28.41 | 1,44 | 1.41E-02 |
| organonitrogen compound metabolic process (GO:1901564) | 724 | 272 | 192.20 | 1,42 | 5.70E-10 |
| cellular metabolic process (GO:0044237) | 1232 | 455 | 327.06 | 1,39 | 3.05E-17 |
| nitrogen compound metabolic process (GO:0006807) | 1032 | 376 | 273.96 | 1,37 | 1.58E-12 |
| RNA processing (GO:0006396) | 103 | 37 | 27.34 | 1,35 | 4.27E-02 |
| carbohydrate metabolic process (GO:0005975) | 148 | 53 | 39.29 | 1,35 | 1.91E-02 |
| primary metabolic process (GO:0044238) | 1106 | 393 | 293.61 | 1,34 | 1.21E-11 |
| organic substance metabolic process (GO:0071704) | 1249 | 441 | 331.57 | 1,33 | 4.31E-13 |
| phosphate-containing compound metabolic process (GO:0006796) | 325 | 114 | 86.28 | 1,32 | 1.58E-03 |
| phosphorus metabolic process (GO:0006793) | 337 | 118 | 89.46 | 1,32 | 1.39E-03 |
| carboxylic acid metabolic process (GO:0019752) | 312 | 109 | 82.83 | 1,32 | 2.27E-03 |
| metabolic process (GO:0008152) | 1391 | 480 | 369.27 | 1,3 | 6.14E-13 |
| oxoacid metabolic process (GO:0043436) | 317 | 109 | 84.15 | 1,3 | 3.69E-03 |
| organic acid metabolic process (GO:0006082) | 319 | 109 | 84.68 | 1,29 | 4.44E-03 |
| small molecule metabolic process (GO:0044281) | 514 | 175 | 136.45 | 1,28 | 3.47E-04 |
| regulation of metabolic process (GO:0019222) | 269 | 88 | 71.41 | 1,23 | 2.68E-02 |
| cellular process (GO:0009987) | 1874 | 608 | 497.49 | 1,22 | 1.61E-12 |
| regulation of cellular metabolic process (GO:0031323) | 254 | 82 | 67.43 | 1,22 | 4.10E-02 |
| regulation of macromolecule metabolic process (GO:0060255) | 264 | 85 | 70.08 | 1,21 | 4.00E-02 |
| cellular response to stimulus (GO:0051716) | 258 | 51 | 68.49 | 0,74 | 1.39E-02 |
| response to stimulus (GO:0050896) | 328 | 64 | 87.07 | 0,74 | 4.31E-03 |
| cell communication (GO:0007154) | 175 | 27 | 46.46 | 0,58 | 1.15E-03 |
| response to chemical (GO:0042221) | 117 | 18 | 31.06 | 0,58 | 7.33E-03 |
| peptidyl-amino acid modification (GO:0018193) | 68 | 10 | 18.05 | 0,55 | 2.85E-02 |
| phosphorelay signal transduction system (GO:0000160) | 97 | 14 | 25.75 | 0,54 | 7.99E-03 |
| intracellular signal transduction (GO:0035556) | 97 | 14 | 25.75 | 0,54 | 7.99E-03 |
| amino acid transport (GO:0006865) | 46 | 6 | 12.21 | 0,49 | 3.98E-02 |
| signal transduction (GO:0007165) | 157 | 19 | 41.68 | 0,46 | 5.30E-05 |
| organic acid transport (GO:0015849) | 58 | 7 | 15.40 | 0,45 | 1.38E-02 |
| signaling (GO:0023052) | 158 | 19 | 41.94 | 0,45 | 4.53E-05 |
| carboxylic acid transport (GO:0046942) | 42 | 5 | 11.15 | 0,45 | 3.36E-02 |
| response to external stimulus (GO:0009605) | 94 | 11 | 24.95 | 0,44 | 1.31E-03 |
| cell adhesion (GO:0007155) | 30 | 3 | 7.96 | 0,38 | 4.28E-02 |
| organic acid transmembrane transport (GO:1903825) | 31 | 3 | 8.23 | 0,36 | 3.57E-02 |
| carboxylic acid transmembrane transport (GO:1905039) | 31 | 3 | 8.23 | 0,36 | 3.57E-02 |
| negative regulation of bacterial-type flagellum-dependent cell motility (GO:1902201) | 24 | 2 | 6.37 | 0,31 | 4.68E-02 |
| protein phosphorylation (GO:0006468) | 48 | 4 | 12.74 | 0,31 | 4.32E-03 |
| cell-substrate adhesion (GO:0031589) | 24 | 2 | 6.37 | 0,31 | 4.68E-02 |
| single-species submerged biofilm formation (GO:0090609) | 24 | 2 | 6.37 | 0,31 | 4.68E-02 |
| submerged biofilm formation (GO:0090605) | 24 | 2 | 6.37 | 0,31 | 4.68E-02 |
| cell adhesion involved in single-species biofilm formation (GO:0043709) | 24 | 2 | 6.37 | 0,31 | 4.68E-02 |
| cell adhesion involved in biofilm formation (GO:0043708) | 24 | 2 | 6.37 | 0,31 | 4.68E-02 |
| negative regulation of cell motility (GO:2000146) | 24 | 2 | 6.37 | 0,31 | 4.68E-02 |
| regulation of cell motility (GO:2000145) | 24 | 2 | 6.37 | 0,31 | 4.68E-02 |
| negative regulation of cellular component movement (GO:0051271) | 24 | 2 | 6.37 | 0,31 | 4.68E-02 |
| regulation of cellular component movement (GO:0051270) | 24 | 2 | 6.37 | 0,31 | 4.68E-02 |
| regulation of bacterial-type flagellum-dependent cell motility (GO:1902021) | 24 | 2 | 6.37 | 0,31 | 4.68E-02 |
| negative regulation of locomotion (GO:0040013) | 25 | 2 | 6.64 | 0,3 | 3.85E-02 |
| regulation of locomotion (GO:0040012) | 26 | 2 | 6.90 | 0,29 | 3.15E-02 |
| localization of cell (GO:0051674) | 29 | 2 | 7.70 | 0,26 | 1.71E-02 |
| cell motility (GO:0048870) | 29 | 2 | 7.70 | 0,26 | 1.71E-02 |
| movement of cell or subcellular component (GO:0006928) | 29 | 2 | 7.70 | 0,26 | 1.71E-02 |
| locomotion (GO:0040011) | 86 | 5 | 22.83 | 0,22 | 6.74E-06 |
| transition metal ion homeostasis (GO:0055076) | 19 | 1 | 5.04 | 0,2 | 3.86E-02 |
| peptidyl-histidine phosphorylation (GO:0018106) | 39 | 2 | 10.35 | 0,19 | 2.00E-03 |
| peptidyl-histidine modification (GO:0018202) | 39 | 2 | 10.35 | 0,19 | 2.00E-03 |
| taxis (GO:0042330) | 64 | 3 | 16.99 | 0,18 | 3.71E-05 |
| chemotaxis (GO:0006935) | 64 | 3 | 16.99 | 0,18 | 3.71E-05 |
| bacterial-type flagellum-dependent cell motility (GO:0071973) | 24 | 1 | 6.37 | 0,16 | 1.24E-02 |
| cilium or flagellum-dependent cell motility (GO:0001539) | 25 | 1 | 6.64 | 0,15 | 9.85E-03 |
| archaeal or bacterial-type flagellum-dependent cell motility (GO:0097588) | 25 | 1 | 6.64 | 0,15 | 9.85E-03 |
