# Supplementary material for: Nonessential tRNA and rRNA modifications impact the bacterial response to sub-MIC antibiotic stress
Source: Microlife. 2022 Sep 14;3:uqac019. doi: 10.1093/femsml/uqac019 (PMC10117853; doi:10.1093/femsml/uqac019)
Supplement: uqac019_Supplemental_Files [file uqac019_supplemental_files.zip › TableS3_18august2022_supplementary_data.docx]

**Table S3. Strains and plasmids**

| **Strain** | **Strain number** | **Construction** |
| --- | --- | --- |
| ***Vibrio cholerae*** | | |
| N16961 hapR+ wt strain | F606 | Gift from Melanie Blokesch |
| N16961 hapR+ wt strain *∆lacZ* | K329 | deletion of lacZ by plasmid integration and excision by sucrose counterselection as described |
| *∆tgt (VC0741)* | J420 | PCR amplification of 500bp up and down regions of VC0741 using primers ZIP431/432 and ZIP433/434. PCR amplicifaction of aadA7 conferring spectinomycin resistance on pAM34 using ZB47/48. PCR assembly of the VC0741::spec fragment using ZIP431/434 and allelic exchange by natural transformation. |
| *∆tgt (VC0741)* | M087 | allelic exchange by integration and excision of conjugative suicide plasmid pMP7 L910, replacing the gene with frt::kan::frt as described previously (Val et al PLoS Genetics 2012, Negro et al, mBio 2019) |
| *∆gidA (VC2775)* | H244 | PCR amplification of 500bp up and down regions of VC2775 using primers ZIP316/317 and ZIP318/319. PCR amplicifaction of aadA7 conferring spectinomycin resistance on pAM34 using ZIP320/321. PCR assembly of the VC2775::spec fragment using ZIP316/319 and allelic exchange by natural transformation. |
| *∆dusA (VC0379)* | L607 | allelic exchange by integration and excision of conjugative suicide plasmid pMP7 L024, replacing the gene with frt::kan::frt |
| *∆dusB (VC0291)* | L606 | allelic exchange by integration and excision of conjugative suicide plasmid pMP7 L416, replacing the gene with frt::kan::frt |
| *∆miaB (VC0962)* | K013 | (Negro et al, mBio 2019) |
| *∆truA (VC0999)* | N095 | allelic exchange by integration and excision of conjugative suicide plasmid pMP7 970, replacing the gene with frt::kan::frt |
| *∆truB (VC0645)* | M562 | allelic exchange by integration and excision of conjugative suicide plasmid pMP7 M347, replacing the gene with frt::kan::frt |
| *∆truC (VC0888)* | P638 | allelic exchange by integration and excision of conjugative suicide plasmid pMP7 O651, replacing the gene with frt::kan::frt |
| *∆trmA (VC0154)* | M564 | allelic exchange by integration and excision of conjugative suicide plasmid pMP7 M423, replacing the gene with frt::kan::frt |
| *∆trmB (VC0453)* | M096 | allelic exchange by integration and excision of conjugative suicide plasmid pMP7 L974, replacing the gene with frt::kan::frt |
| *∆trmE (VC0003)* | H218 | PCR amplification of 500bp up and down regions of VC0003 using primers 1640/1641 and 1642/1643. PCR amplicifaction of aadA7 conferring spectinomycin resistance on pAM34 using 1644/1645. PCR assembly of the VC0003::spec fragment using 1640/1643 and allelic exchange by natural transformation. |
| *∆trmH (VC0803)* | Q062 | allelic exchange by integration and excision of conjugative suicide plasmid pMP7 P493, replacing the gene with frt::kan::frt |
| *∆trmK VCA0634)* | K650 | allelic exchange by integration and excision of conjugative suicide plasmid pMP7 K440, replacing the gene with frt::kan::frt |
| *∆rlmN (VC0757)* | M094 | allelic exchange by integration and excision of conjugative suicide plasmid pMP7 L912, replacing the gene with frt::kan::frt |
| *∆rlmI (VC1354)* | N031 | allelic exchange by integration and excision of conjugative suicide plasmid pMP7 M969, replacing the gene with frt::kan::frt |
| *∆rsuA (VC1635)* | H497 | Negro et al, mBio 2019 |
| *∆rsmB (VC0044)* | N033 | allelic exchange by integration and excision of conjugative suicide plasmid pMP7 M771, replacing the gene with frt::kan::frt |
| *∆rsmC (VC0623)* | L601 | allelic exchange by integration and excision of conjugative suicide plasmid pMP7 L577, replacing the gene with frt::kan::frt |
| *∆rsmD (VC0146)* | M088 | allelic exchange by integration and excision of conjugative suicide plasmid pMP7 L565, replacing the gene with frt::kan::frt |
| *∆rsmF (VC2223)* | N045 | allelic exchange by integration and excision of conjugative suicide plasmid pMP7 M769, replacing the gene with frt::kan::frt |
| *∆rluB (VC1179)* | L559 | allelic exchange by integration and excision of conjugative suicide plasmid pMP7 L020, replacing the gene with frt::kan::frt |
| *∆rluD (VC0709)* | N097 | allelic exchange by integration and excision of conjugative suicide plasmid pMP7 N035, replacing the gene with frt::kan::frt |
| *∆rluE (VC1140)* | Q061 | allelic exchange by integration and excision of conjugative suicide plasmid pMP7 P346, replacing the gene with frt::kan::frt |
| ***Escherichia coli*** | | |
| MG1655 wt strain |  | laboratory collection |
| *∆tgt* | J233 | P1 transduction from KEIO strain JW0396-3 |
| *∆gidA* | J193 | P1 transduction from KEIO strain JW3719-1 |
| *∆dusA* | J196 | P1 transduction from KEIO strain JW5950-5 |
| *∆dusB* | J243 | P1 transduction from KEIO strain JW3228-1 |
| *∆trmE* | J194 | P1 transduction from KEIO strain JW3684-1 |
| *∆rsuA* | H243 | P1 transduction from KEIO strain JW2171-1 |
| *∆rsmC* | J192 | P1 transduction from KEIO strain JW4333-1 |
| *∆rsmD* | J241 | P1 transduction from KEIO strain JW3430-4 |
| *∆rluB* | J235 | P1 transduction from KEIO strain JW1261-3 |
| **Plasmids pMP7-∆gene::kan** | | gibson assembly using primers MV450/451 for the amplification of pMP7 vector, primers indicated below for up and down regions of the gene, and primers MV268/269 on pKD4 plasmid for the resistance gene (frt::kan::frt). |
| *∆tgt (VC0741)* | L910 | VC0741tgt5/7 for up region and VC0741tgt6bis/8 bis for down region |
| *∆dusA (VC0379)* | L024 | VC0379dusA5/7 for up region and VC0379dusA6/8 for down region |
| *∆dusB (VC0291)* | L416 | VC0291dusB5/7 for up region and VC0291dusB6bis/8 bis for down region |
| *∆truA (VC0999)* | M970 | VC0999truA5/7 for up region and VC0999truA6bis/8bis for down region |
| *∆truB (VC0645)* | M347 | VC0645truB5bis/7bis for up region and VC0645truB6/8 for down region |
| *∆truC (VC0888)* | O651 | VC0888truC5/7 for up region and VC0888truC6/8 for down region |
| *∆trmA (VC0154)* | M423 | VC0154trmA5/7 for up region and VC0154trmA6bis/8 bis for down region |
| *∆trmB (VC0453)* | L974 | VC0453trmB5/7 for up region and VC0453trmB6/8 for down region |
| *∆trmH (VC0803)* | P493 | VC0803trmH5/7 for up region and VC0803trmH6bis/8 for down region |
| *∆trmK VCA0634)* | K440 | VCA06345/7 for up region and VCA06346/8 for down region |
| *∆rlmN (VC0757)* | L912 | VC0757rlmN5/7 for up region and VC0757rlmN6/8 for down region |
| *∆rlmI (VC1354)* | M969 | VC1354rlmI5bis/7bis for up region and VC1354rlmI6/8 for down region |
| *∆rsmB (VC0044)* | M771 | VC0044rsmB5/7 for up region and VC0044rsmB6/8 for down region |
| *∆rsmC (VC0623)* | L577 | VC0623rsmC5/7 for up region and VC0623rsmC6/8 for down region |
| *∆rsmD (VC0146)* | L565 | VC0146rsmD5/7 for up region and VC0146rsmD6/8 for down region |
| *∆rsmF (VC2223)* | M769 | VC1502rsmF5/7 for up region and VC1502rsmF6/8 for down region |
| *∆rluB (VC1179)* | L020 | VC1179rluB5/7 for up region and VC1179rluB6/8 for down region |
| *∆rluD (VC0709)* | N035 | VC0709rluD5ter/7ter for up region and VC0709rluD6bis/8ter for down region |
| *∆rluE (VC1140)* | P346 | VC1140rluE5/7 for up region and VC1140rluE6/8 for down region |
