# Supplementary material for: Nonessential tRNA and rRNA modifications impact the bacterial response to sub-MIC antibiotic stress
Source: Microlife. 2022 Sep 14;3:uqac019. doi: 10.1093/femsml/uqac019 (PMC10117853; doi:10.1093/femsml/uqac019)
Supplement: uqac019_Supplemental_Files [file uqac019_supplemental_files.zip › TableS4_18august2022_supplementary_data.docx]

**Table S4. Primers**

| **primers** |  |
| --- | --- |
| ZB47 | CCCGTTCCATACAGAAGCTGGGCGAACAAACGATGCTCGC |
| ZB48 | GACATTATTTGCCGACTACCTTGGTGATCTCGCCTTTCACG |
| ZIP431 | CACCTCAGTACGTTCACTCG |
| ZIP432 | GCGAGCATCGTTTGTTCGCCCAGCTTCTGTATGGAACGGGGAAGCCTCCAATGTCAGAGAAACAGTCTGACC |
| ZIP433 | CGTGAAAGGCGAGATCACCAAGGTAGTCGGCAAATAATGTCTGATTTCGTGCACTGGGTTGGATTTGC |
| ZIP434 | CTGCAACGGCGATGGTTAACATCACC |
| ZIP316 | GGCTCAAACGCGTCATCAATCG |
| ZIP317 | GTTCTGGACCAGTTGCGTGAGCGCATGAACGACCTCAGGTATTCATCG |
| ZIP318 | GAAAGGCGAGATCACCAAGGTAGTCGGCAAATAATGAATCCGTTACGAGTCAAACTCG |
| ZIP319 | GGCTAAAAATACCCCAGCATTCGG |
| ZIP320 | CCCATAGTCTCGATGAATACCTGAGGTCGTTCATGCGCTCACGCAACTGG |
| ZIP321 | AGGCGTCGAGTTTGACTCGTAACGGATTCATTATTTGCCGACTACCTTGGTGATCTCG |
| 1640 | TCCACAATCCAAAAAAAAGAGCGGCATTAAGCCGC |
| 1641 | GCGAGCATCGTTTGTTCGCCCAGCTTCTGTATGGAACGGGGTAACGCTATGATTCATATCATTACTGGCAGC |
| 1642 | CGTGAAAGGCGAGATCACCAAGGTAGTCGGCAAATAATGTCTGTAATCAGCAGCCAACAAAAAGGCGACCTTTTGG |
| 1643 | ATGCGTGAGCGGATTGGCGATGATCGCCAGCGC |
| 1644 | CCGCACCGCCTAGGGTGCTGCCAGTAATGATATGAATCATAGCGTTACTCCCGTTCCATACAGAAGCTGGGCGAACAAACGATGCTCGC |
| 1645 | AGAGGCGGCCAAAAGGTCGCCTTTTTGTTGGCTGCTGATTACAGACATTATTTGCCGACTACCTTGGTGATCTCGCCTTTCACG |
| VC0741tgt5 | CTATTATTTAAACTCTTTCCgtgcggcgcaagatgcgctg |
| VC0741tgt7 | CTACACAATCGCTCAAGACGTGaagcctccaatgtcagaga |
| VC0741tgt8bis | CTAATTCCCATGTCAGCCGTTGCGGTGGGTGAGCCAAAAG |
| VC0741tgt6bis | TACGTAGAATGTATCAGACTGTGCACGAAATCAGGCTTTG |
| VC0379dusA5 | CTATTATTTAAACTCTTTCCagcagttcatacgcacttgat |
| VC0379dusA7 | CTACACAATCGCTCAAGACGTGaggtgaacgtccccatttca |
| VC0379dusA8 | CTAATTCCCATGTCAGCCGTaaaacaccaaacattagcgaaaatg |
| VC0379dusA6 | TACGTAGAATGTATCAGACTcagtgcggtgactagcgccg |
| VC0291dusB5 | CTATTATTTAAACTCTTTCCgccatcaaacttggtgccg |
| VC0291dusB7 | CTACACAATCGCTCAAGACGTGaacgtcttcacagcttcgtac |
| VC0291dusB8bis | CTAATTCCCATGTCAGCCGTTAGCCGAAGACTGCGGCATA |
| VC0291dusB6bis | TACGTAGAATGTATCAGACTcggtctagctcttctcttaaTT |
| VC0999truA5 | CTATTATTTAAACTCTTTCCGGAGCCGCAAGCGAAGCCTG |
| VC0999truA7 | CTACACAATCGCTCAAGACGTGCTGTTTGTCTCAATAAAAATGG |
| VC0999truA8bis | CTAATTCCCATGTCAGCCGTCGATTCAGTGCCACTGCACG |
| VC0999truA6bis | TACGTAGAATGTATCAGACTTAGTTCAAATTATCAGGCAAG |
| VC0645truB5bis | CTATTATTTAAACTCTTTCCGAATCTTGTCTGGCAGCATTG |
| VC0645truB7bis | CTACACAATCGCTCAAGACGTGCGTTTGTACCACTTGACCATC |
| VC0645truB8 | CTAATTCCCATGTCAGCCGTattcgtggaagggctcgctg |
| VC0645truB6 | TACGTAGAATGTATCAGACTgtacaaactgtgctttgcagc |
| VC0888truC5 | CTATTATTTAAACTCTTTCCGGCGGTTATCCATCGCTTGC |
| VC0888truC7 | CTACACAATCGCTCAAGACGTGCTATTGGCACACCTTGTCGA |
| VC0888truC8 | CTAATTCCCATGTCAGCCGTGATTTTCTCAATCAGCCCA |
| VC0888truC6 | TACGTAGAATGTATCAGACTAATCGGCAAAGGCCGCTTAA |
| VC0154trmA5 | CTATTATTTAAACTCTTTCCgcttcgttggcgtgtaaataa |
| VC0154trmA7 | CTACACAATCGCTCAAGACGTGtcgggtacctttttatgggc |
| VC0154trmA8bis | CTAATTCCCATGTCAGCCGTGGGCGGTCGATTGTACTCAA |
| VC0154trmA6bis | TACGTAGAATGTATCAGACTgcggTTACTTTTTGCGCTCT |
| VC0453trmB5 | CTATTATTTAAACTCTTTCCagattgcgcgctctggcgt |
| VC0453trmB7 | CTACACAATCGCTCAAGACGTGttctattgcctgcacattactaaa |
| VC0453trmB8 | CTAATTCCCATGTCAGCCGTtttgaccctaatcctcaagc |
| VC0453trmB6 | TACGTAGAATGTATCAGACTagagaggcgactttgcagca |
| VC0803trmH5 | CTATTATTTAAACTCTTTCCAAAAGCTTAGAACTGTTGTTGC |
| VC0803trmH7 | CTACACAATCGCTCAAGACGTGGTGCTGTCCGAGATAAATG |
| VC0803trmH8 | CTAATTCCCATGTCAGCCGTTAATCCATTTACCTAAAACGAAT |
| VC0803trmH6bis | TACGTAGAATGTATCAGACTTATCGAACTCAACGCTGAG |
| VCA06345 | CTATTATTTAAACTCTTTCCaagtgcagcaaataccagcc |
| VCA06347 | CTACACAATCGCTCAAGACGTGctgtacctacatgcggtaa |
| VCA06348 | CTAATTCCCATGTCAGCCGTgctagaaagatgaaagcactc |
| VCA06346 | TACGTAGAATGTATCAGACTcgctcgttagaatcgcacag |
| VC0757rlmN5 | CTATTATTTAAACTCTTTCCattgccgcgaaaatggttcac |
| VC0757rlmN7 | CTACACAATCGCTCAAGACGTGgtgcctctcaagaagacgg |
| VC0757rlmN8 | CTAATTCCCATGTCAGCCGTttttgcctaatatggcaacaaatc |
| VC0757rlmN6 | TACGTAGAATGTATCAGACTggttctcttcaatgctttgga |
| VC1354rlmI5bis | CTATTATTTAAACTCTTTCCGCATAGCCGAAATCCACGTT |
| VC1354rlmI7bis | CTACACAATCGCTCAAGACGTGCTTGATATTTGTCGATAGTAATG |
| VC1354rlmI8 | CTAATTCCCATGTCAGCCGTGCGATGGCGTGAGCGAACGT |
| VC1354rlmI6 | TACGTAGAATGTATCAGACTCAAACAAGCCTTTTCTCG |
| VC0044rsmB5 | CTATTATTTAAACTCTTTCCGTACGACAAACTGGCAGAAC |
| VC0044rsmB7 | CTACACAATCGCTCAAGACGTGCCGACGCAGCAGCGGCGCG |
| VC0044rsmB8 | CTAATTCCCATGTCAGCCGTTTCTATTACGCCGTATTAAGC |
| VC0044rsmB6 | TACGTAGAATGTATCAGACTCACCACTTGCAGCGCGCCC |
| VC0623rsmC5 | CTATTATTTAAACTCTTTCCttttttaaccgccatcgcg |
| VC0623rsmC7 | CTACACAATCGCTCAAGACGTGacacgctctactcaagaaaa |
| VC0623rsmC8 | CTAATTCCCATGTCAGCCGTctcgttacattttgccgcttt |
| VC0623rsmC6 | TACGTAGAATGTATCAGACTggaaagcattcaccagacc |
| VC0146rsmD5 | CTATTATTTAAACTCTTTCCttgttcaacaatacgtgcagg |
| VC0146rsmD7 | CTACACAATCGCTCAAGACGTGagattttttgaccgctaattaagtg |
| VC0146rsmD8 | CTAATTCCCATGTCAGCCGTaagcactactctggttagcga |
| VC0146rsmD6 | TACGTAGAATGTATCAGACTggtgaactgtggctacaagag |
| VC1502rsmF5 | CTATTATTTAAACTCTTTCCAGAGTGAAAAACGCGGCTCG |
| VC1502rsmF7 | CTACACAATCGCTCAAGACGTGAAGCGAGTTCCGTAGATGAAT |
| VC1502rsmF8 | CTAATTCCCATGTCAGCCGTTGGCTGAACTATAGTATTAAATTC |
| VC1502rsmF6 | TACGTAGAATGTATCAGACTCATGATTTTCTCCTCAGATGTC |
| VC1179rluB5 | CTATTATTTAAACTCTTTCCgtttcgtttgttgaaaaacaata |
| VC1179rluB7 | CTACACAATCGCTCAAGACGTGttatctacctatgtgtcgtctt |
| VC1179rluB8 | CTAATTCCCATGTCAGCCGTgctgtttattgtttgaagctg |
| VC1179rluB6 | TACGTAGAATGTATCAGACTcaatacttggcttggcga |
| VC0709rluD5ter | CTATTATTTAAACTCTTTCCATAGGTGCGTAAACCGCGCGC |
| VC0709rluD7ter | CTACACAATCGCTCAAGACGTGCGATAATGTCGTCATCTTCATA |
| VC0709rluD8ter | CTAATTCCCATGTCAGCCGTCTCGACCAAACGTACTTTAA |
| VC0709rluD6bis | TACGTAGAATGTATCAGACTTAATACTCTTCAGTGCGGTT |
| VC1140rluE5 | CTATTATTTAAACTCTTTCCTGATACGTTGTTCTTCGGTC |
| VC1140rluE7 | CTACACAATCGCTCAAGACGTGTTAAGCACCTTGCAAAAATGT |
| VC1140rluE8 | CTAATTCCCATGTCAGCCGTACTCTGATTAATCAAAAATATATAG |
| VC1140rluE6 | TACGTAGAATGTATCAGACTGATGCACAAGAGGAGTCACA |
