# Supplementary figures and images for: Nonessential tRNA and rRNA modifications impact the bacterial response to sub-MIC antibiotic stress
Source: Microlife. 2022 Sep 14;3:uqac019. doi: 10.1093/femsml/uqac019 (PMC10117853; doi:10.1093/femsml/uqac019)

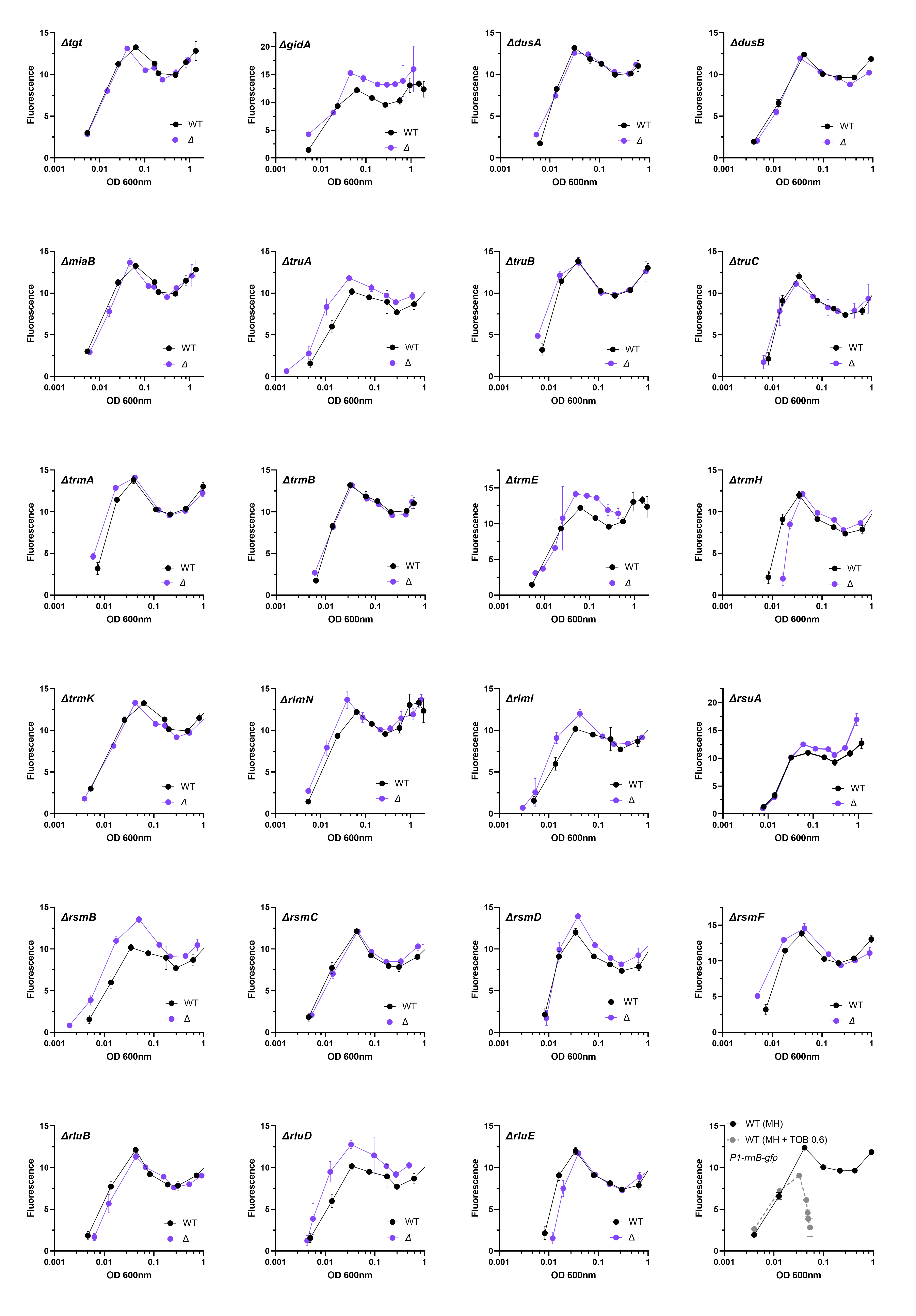

Supplement: uqac019_Supplemental_Files [file uqac019_supplemental_files.zip › Figure_S1_stringent_response_supplementary_data.tif]

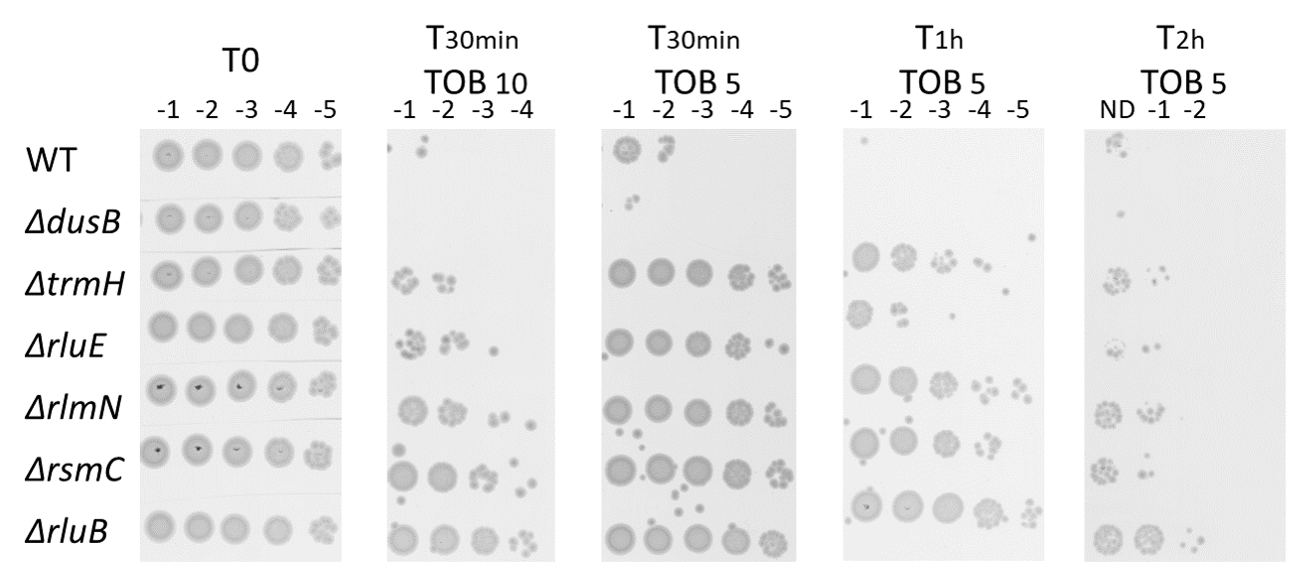

Supplement: uqac019_Supplemental_Files [file uqac019_supplemental_files.zip › Figure_S2_survival_spots_supplementary_data.tif]

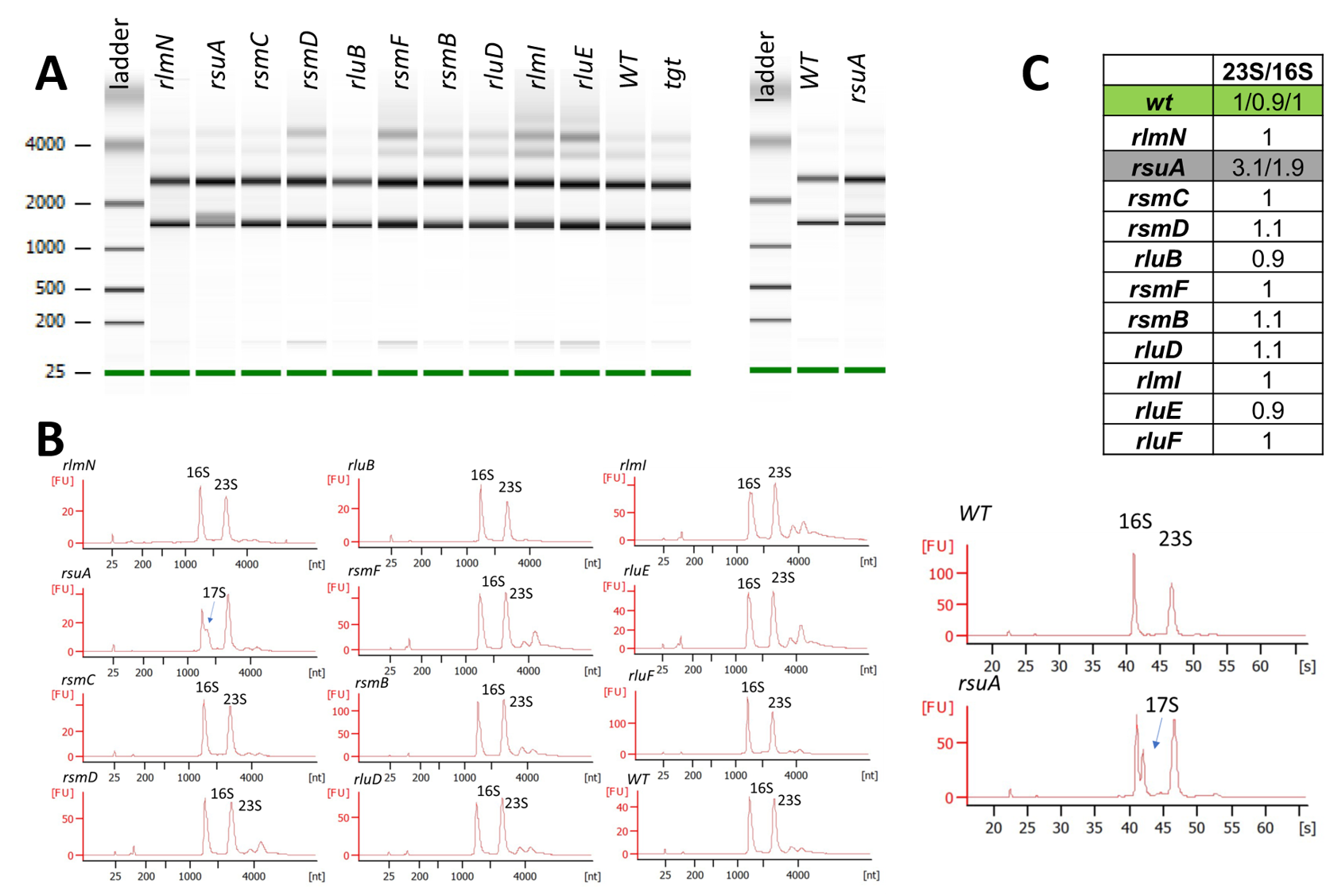

Supplement: uqac019_Supplemental_Files [file uqac019_supplemental_files.zip › Figure_S3_17S_supplementary_data.tif]

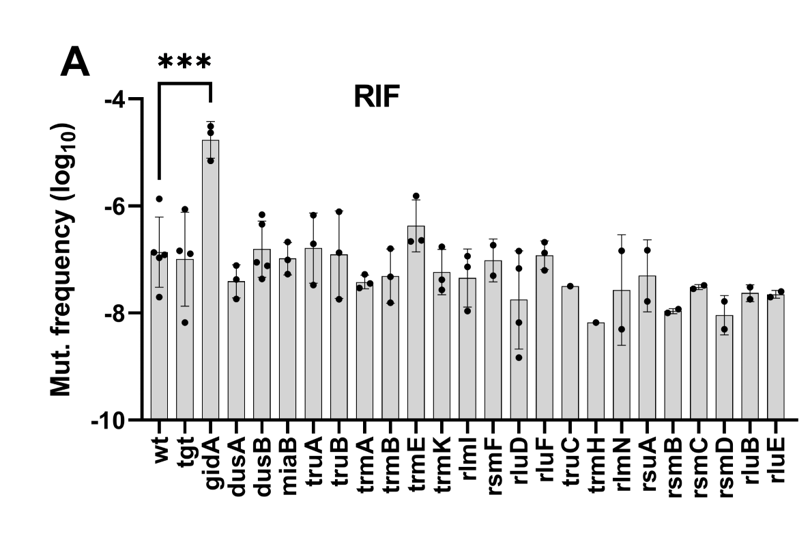

Supplement: uqac019_Supplemental_Files [file uqac019_supplemental_files.zip › Figure_S4_mutations_supplementary_data.tif]

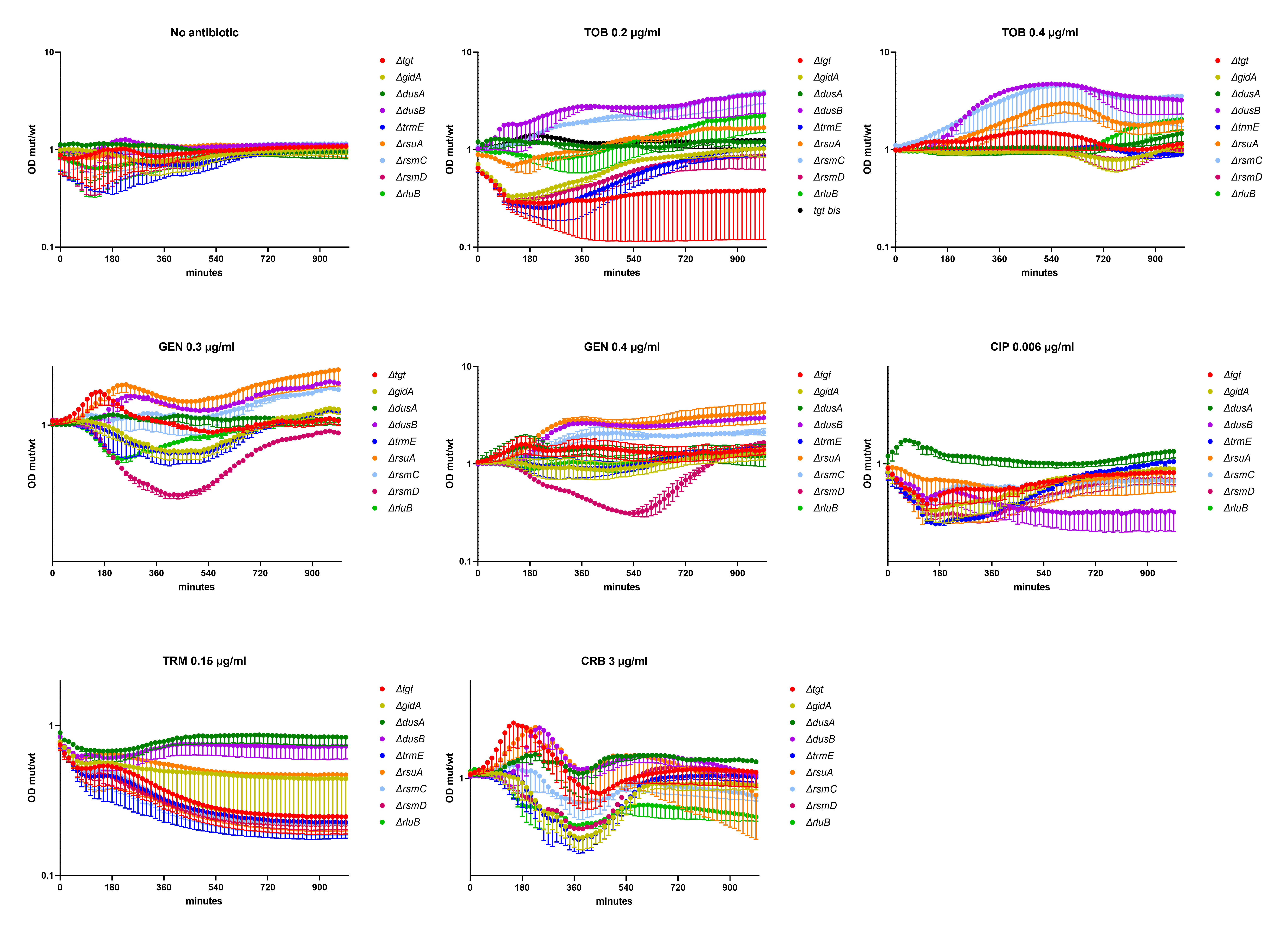

Supplement: uqac019_Supplemental_Files [file uqac019_supplemental_files.zip › Figure_S5_MG1655_supplementary_data.tif]

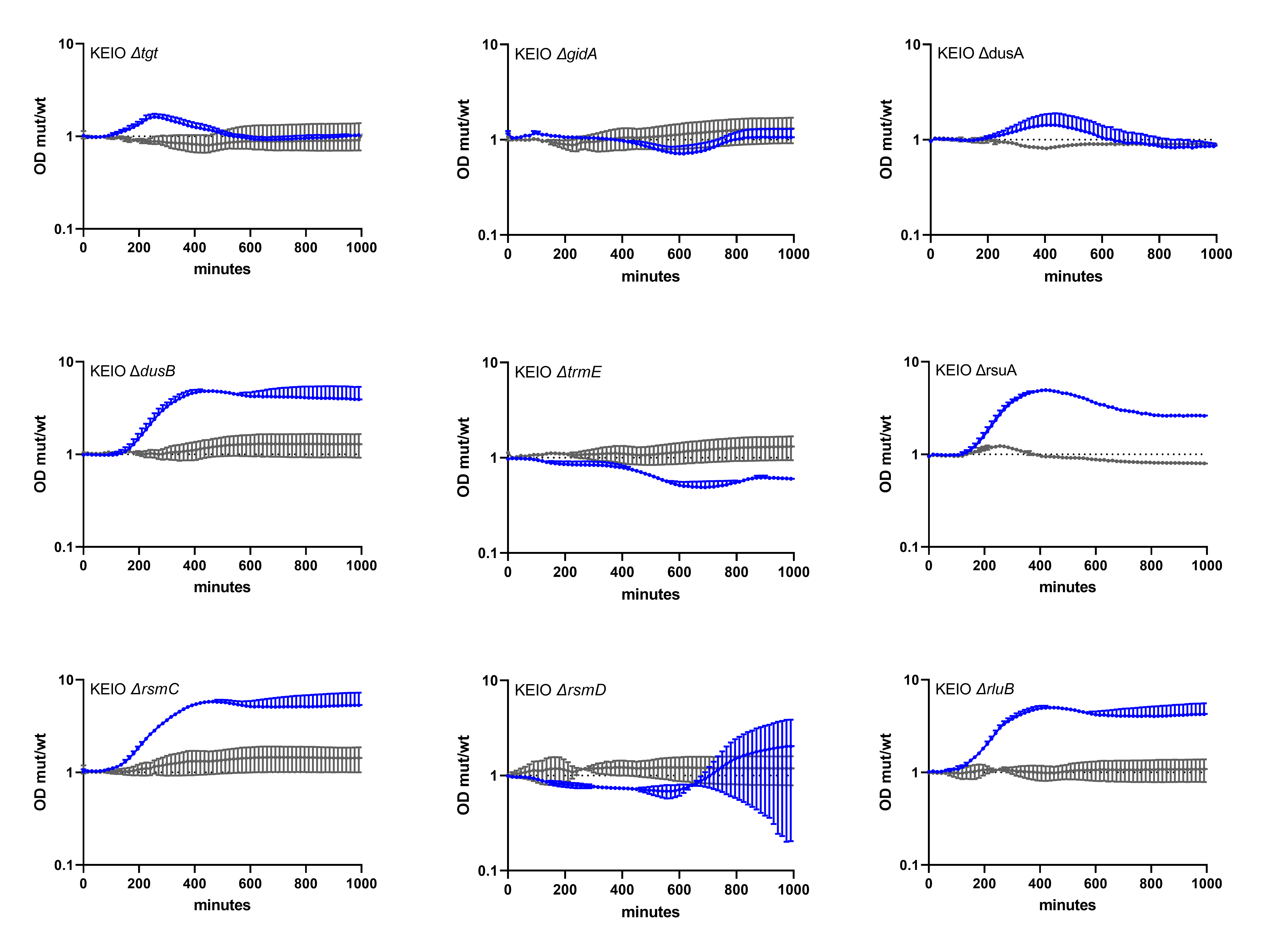

Supplement: uqac019_Supplemental_Files [file uqac019_supplemental_files.zip › Figure_S6_keio_supplementary_data.tif]
